# Integrating mTOR Inhibition and Photodynamic Therapy Based on Carrier‐Free Nanodrugs for Breast Cancer Immunotherapy

**DOI:** 10.1002/adhm.202402357

**Published:** 2024-09-05

**Authors:** Jinzhao Liu, Qingyang Lyu, Meicen Wu, Yang Zhou, Tianyi Wang, Yichi Zhang, Ni Fan, Chang Yang, Weiping Wang

**Affiliations:** ^1^ State Key Laboratory of Pharmaceutical Biotechnology The University of Hong Kong Hong Kong 999077 China; ^2^ Department of Pharmacology and Pharmacy Li Ka Shing Faculty of Medicine The University of Hong Kong Hong Kong 999077 China; ^3^ Dr. Li Dak‐Sum Research Centre The University of Hong Kong Hong Kong 999077 China

**Keywords:** anti‐angiogenesis, carrier‐free nanoparticles, immunogenic cell death, mTOR inhibition, photodynamic therapy, synergistic effect

## Abstract

Conventional photodynamic therapy (PDT) in cancer treatment needs to utilize oxygen to produce reactive oxygen species to eliminate malignant tissues. However, oxygen consumption in tumor microenvironment exacerbates cancer cell hypoxia and may promote vasculature angiogenesis. Since the mammalian target of rapamycin (mTOR) signaling pathway plays a vital role in endothelial cell proliferation and fibrosis, mTOR inhibitor drugs hold the potential to reverse hypoxia‐evoked angiogenesis for improved PDT effect. In this study, a carrier‐free nanodrug formulation composed of Torin 1 as mTORC1/C2 dual inhibitor and Verteporfin as a photosensitizer and Yes‐associated protein inhibitor is developed. These two drug molecules can self‐assemble into stable nanoparticles through *π–π* stacking and hydrophobic interactions with good long‐term stability. The nanodrugs can prompt synergistic apoptosis, combinational anti‐angiogenesis, and strong immunogenic cell death effects upon near‐infrared light irradiation in vitro. Furthermore, the nanosystem also exhibits improved antitumor effect, anti‐cancer immune response, and distant tumor inhibition through tumor microenvironment remodeling in vivo. In this way, the nanodrugs can reverse PDT‐elicited angiogenesis and promote cancer immunotherapy to eliminate tumor tissues and prevent metastasis. This nanosystem provides insights into integrating mTOR inhibitors and photosensitizers for safe and effective breast cancer treatment in clinical settings.

## Graphical abstract

Carrier‐free nanodrugs composed of photosensitizer Verteporfin and mammalian target of rapamycin inhibitor Torin 1 are designed for breast cancer treatment with improved photodynamic therapy and tumor microenvironment reshaping effects. The nanodrugs can promote synergistic apoptosis, combinational anti‐angiogenesis, and strong immunogenic cell death effects within tumor tissues upon light irradiation, which provides novel insights for safe and efficient breast cancer immunotherapy.

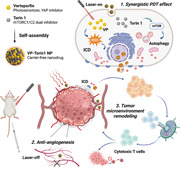

## 1 Introduction

Metastasis is one of the leading reasons for cancer recurrence, treatment failure, and poor prognosis, especially in triple‐negative breast cancer patients.^[^ ^1^ ^]^ Triple‐negative breast cancer cells are characterized with multiple resistant mechanisms, high invasive nature, and immune evasion properties.^[^ ^2^ ^]^ Therefore, commonly used surgery and radiation therapy are difficult to eradicate tumor tissues and may increase the invasive potential of residue cancer cells.^[^ ^3^ ^]^ For first‐line chemotherapeutics, such as paclitaxel, cisplatin, and cyclophosphamide, they are hard to be delivered into tumor tissues and prone to cause side effects in normal tissues.^[^ ^4^ ^]^ Besides, standard immunotherapy such as anti‐PD‐1/PD‐L1 antibodies also exhibit modest therapeutic effects in ≈70–80% cancer patients, mainly due to the immunologically cold tumor microenvironment.^[^ ^5^ ^]^ One of the promising anti‐cancer strategies is photodynamic therapy (PDT) that utilizes photosensitizers to generate reactive oxygen species (ROS) with the help of oxygen and light irradiation.^[^ ^6^ ^]^ Using an external light source as the trigger, PDT can produce cytotoxic ROS and efficiently eliminate cancer cells with spatio‐temporal control, which has been approved for treating certain types of cancers, including esophageal and bladder cancer, by the U.S. Food and Drug Administration (FDA).^[^ ^7^ ^]^ The irradiation location, time, and duration can be easily adjusted in response to pathological states and patient needs in clinical practice.

Owing to the aberrant glycolysis in breast cancer cells, the tumor microenvironment is weakly acidic with low oxygen and nutrient levels.^[^ ^8^ ^]^ However, oxygen is needed for photosensitizers to generate ROS during the PDT process, which may cause PDT‐evoked tumor hypoxia and further lead to vascular angiogenesis and cancer aggressiveness, thus reducing antitumor efficacy.^[^ ^9^ ^]^ To solve this problem, anti‐angiogenesis agents are ideal candidates to overcome the limitations and improve PDT efficacy. A commonly used anti‐angiogenesis drug in clinical practice is systemic sirolimus (Rapamycin) that can inhibit the bioactivity of the mammalian target of rapamycin (mTOR) involved in vascular repair and suppress the endothelial cell differentiation.^[^ ^10^ ^]^ However, the combinational anti‐cancer effect between mTOR inhibitors and photosensitizers for anti‐angiogenesis and improved PDT has not been explored before. In addition, systemic administration of mTOR inhibitor drugs will also cause some side effects, including anemia, mouth sores, gastrointestinal disorders, etc.^[^ ^11^ ^]^ Hence, new therapeutic strategies and drug delivery systems are considerably needed for safe and efficient triple‐negative breast cancer treatment.

Apart from hypoxia‐elicited angiogenesis, the other consideration is the immunosuppressive microenvironment, which is the major cause of metastasis and immune evasion. One of the approaches to improve anti‐cancer immunity is immunogenic cell death (ICD) that alerts the immune system to the presence of dying cancer cells.^[^ ^12^ ^]^ ICD is accompanied by the secretion or exposure of damage‐associated molecular patterns (DAMPs) from apoptotic cancer cells to enhance tumor‐specific antigen presentation, activate the maturation of dendritic cells (DC), and promote immune cell infiltration into tumors.^[^ ^13^ ^]^ It has been reported that PDT can initiate the ICD process by ROS generation and cell apoptosis, which is referred as photoimmunotherapy.^[^ ^14^ ^]^ Besides, mTOR inhibition can also boost ICD in cancer cells through autophagic cell death and adenosine triphosphate (ATP) release.^[^ ^15^ ^]^ Therefore, we hypothesized that the combination between mTOR inhibitor and PDT is promising to remodel the tumor immunosuppressive microenvironment and achieve stronger anti‐cancer immunotherapy to suppress cancer metastasis in breast cancer patients.

Herein, we combined the mTORC1/C2 dual inhibitor Torin 1 and photosensitizer Verteporfin for improved PDT and tumor microenvironment remodeling. Unlike the mTORC1 allosteric inhibitor sirolimus, Torin 1 can bind to the ATP‐binding site of the mTOR kinase domain to inhibit both mTORC1 and mTORC2, thereby avoiding the mTORC2‐mediated hyperactivation of Akt signaling pathways and exerting better anti‐angiogenesis effect.^[^ ^16^ ^]^ Compared with other FDA‐approved photosensitizers, Verteporfin can be activated upon 690 nm near‐infrared (NIR) light irradiation, which is in the phototherapeutic window (650–900 nm) and better than 656 nm (Chlorin e6) and 635 nm (5‐aminolevulinic acid) with excellent biocompatibility and tissue penetration depth.^[^ ^17^ ^]^ Moreover, Verteporfin is also a potent Yes‐associated protein (YAP) inhibitor to suppress pro‐angiogenic and proliferative gene expression, which may contribute to anti‐angiogenesis and anti‐tumorigenesis effects.^[^ ^18^ ^]^

**Figure 1:**
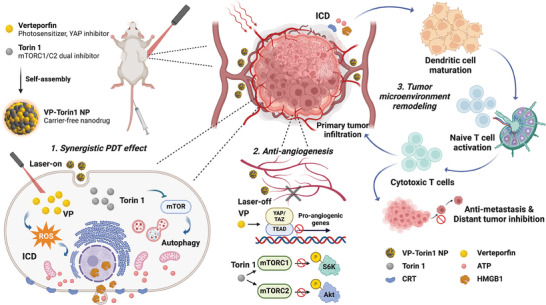
Schematic illustration of improved photodynamic therapy and tumor microenvironment remodeling based on VP‐Torin1 NPs. Upon NIR light irradiation, Verteporfin generates reactive oxygen species and Torin 1 promotes autophagic cell death for synergistic cytotoxicity. Without light irradiation, these two drugs have a combinational anti‐angiogenesis effect to ameliorate hypoxia‐elicited angiogenesis. Nanoparticles can also activate anti‐cancer immunity and inhibit metastasis by photoimmunotherapy to remodel the immunosuppressive microenvironment.

To achieve targeting delivery and avoid side effects, nanoparticle drug delivery systems (NDDS) are promising strategies with better stability, drug solubility, and pharmacokinetics.^[^ ^19^ ^]^ However, most of NDDS need nanocarriers such as liposomes and polymeric micelles to encapsulate drug molecules, which usually have low drug‐loading capacity, complicated preparation process, and are prone to induce an immune response by synthetic materials.^[^ ^20^ ^]^ Interestingly, we found that Verteporfin and Torin 1 could self‐assemble with each other to form carrier‐free nanodrugs without any excipients. The nanoparticles had good stability under physiological conditions and could gradually release two drug molecules under sink conditions. Upon light irradiation, nanoparticles owned higher apoptosis, combinational anti‐angiogenesis, and improved ICD effects in vitro (**Figure** **1**). Additionally, the nanodrugs exhibited effective tumor inhibition and adaptive immune activation effects against 4T1 tumor tissues in vivo. Regarding cancer metastasis, the nanodrugs also exhibited strong distant tumor suppression and abscopal effect based on activated cytotoxic T cells and systemic antitumor immune response. This proof‐of‐concept research will promote the application of the combination of two drug molecules for tumor microenvironment remodeling and facilitate the clinical translation of carrier‐free nanodrugs, ensuring safe and efficient breast cancer treatment.

## 2 Results and Discussion

### 2.1 Synergistic Effect between Verteporfin and Torin 1 in Breast Cancer Cell Lines

**Figure 2:**
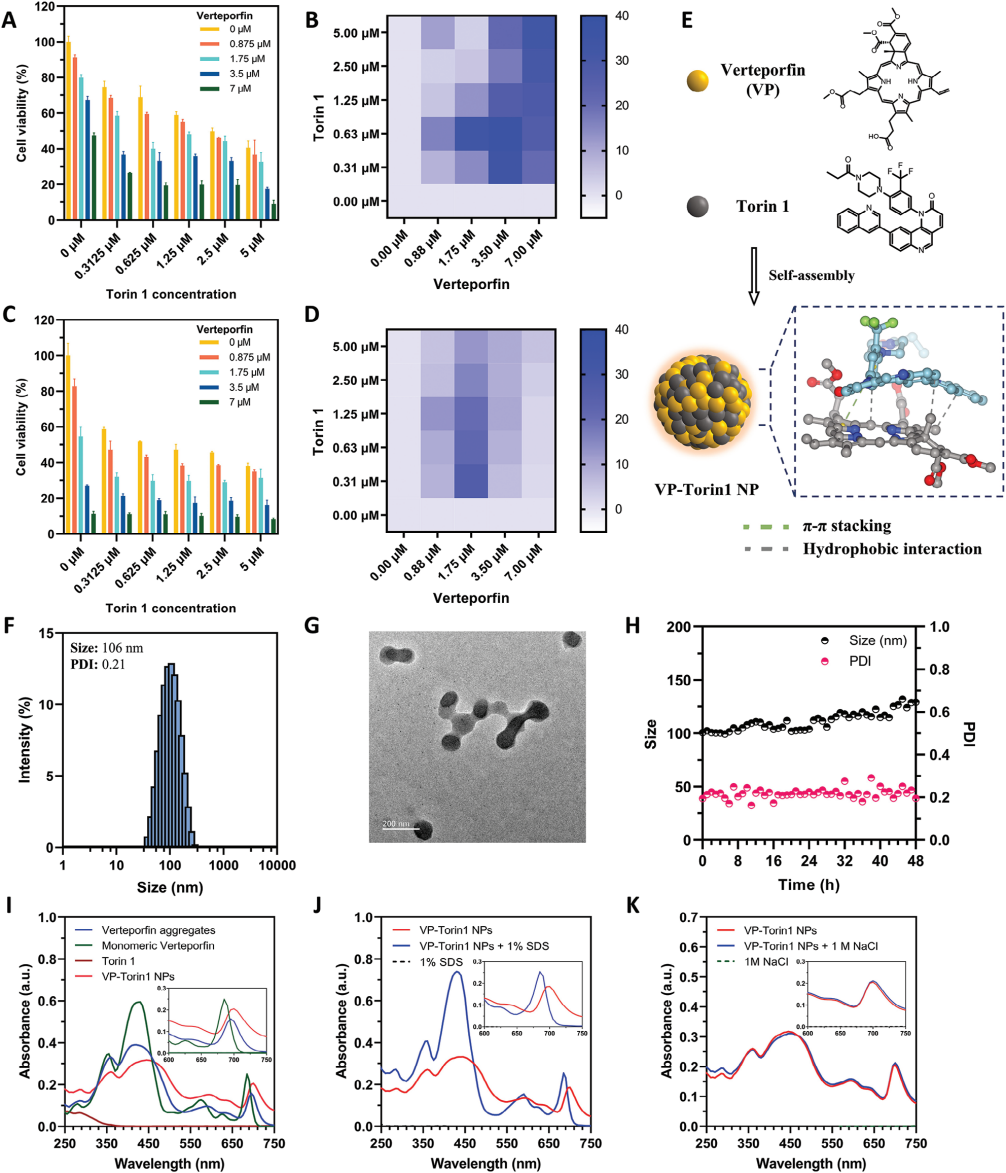
Synergistic effect of Verteporfin and Torin 1 and fabrication/characterization of VP‐Torin1 NPs. Cell viability of 4T1 cells (A) and MDA‐MB‐231 cells (C) after treatment of gradient concentrations of Verteporfin and Torin 1 in the presence of light irradiation (mean ± SD, *n* = 3, Xe lamp, 690 nm, 2.7 mW cm^−^^2^, 1 min). Synergy score of Verteporfin and Torin 1 combination on 4T1 cells (B) and MDA‐MB‐231 cells (D) in the presence of light irradiation calculated by the HSA model in SynergyFinder. E) Schematic illustration of Verteporfin and Torin 1 self‐assembling into carrier‐free nanoparticle formulation through *π–π* stacking (Green line) and hydrophobic interactions (Grey line), predicted by molecular docking analysis. F) Size distribution of VP‐Torin1 NPs after preparation and centrifugation characterized by DLS. G) Transmission electron microscopy (TEM) image of VP‐Torin1 NPs. H) Stability test of VP‐Torin1 NPs in PBS solution at 37 °C for 2 days. I) UV–vis absorption spectra of Verteporfin aggregates (in H_2_O), monomeric Verteporfin (in methanol), Torin 1 (in H_2_O), and VP‐Torin1 NPs (in H_2_O). J) UV–vis absorption spectra of VP‐Torin1 NPs and VP‐Torin1 NPs in the presence of SDS (1%, w/v). K) UV–vis absorption spectra of VP‐Torin1 NPs and VP‐Torin1 NPs in the presence of NaCl (1 m).

First, we chose human triple‐negative breast cancer cell line MDA‐MB‐231 and mouse breast cancer cell line 4T1 to explore the synergistic effect of Verteporfin (photosensitizer) and Torin 1 (mTOR inhibitor). The cancer cells were incubated with gradient concentrations of Verteporfin and Torin 1 for 24 h, followed by light irradiation. 2,5‐diphenyl‐2‐H‐tetrazolium bromide (MTT) assay was conducted to evaluate cell viability after another 24 h. The synergy score was calculated based on cell viability data by the HSA model in the Synergy Finder website.^[^ ^21^ ^]^ For 4T1 cells with light irradiation, Verteporfin (3.5 µm) or Torin 1 (0.625 µm) alone exhibited 67% and 69% viability, respectively (**Figure** **2**A,B). Interestingly, the combinational group with the same concentration showed a significant decrease to 33%. Besides, the average synergy score of combinational groups under light irradiation was ≈20, which demonstrated a strong synergistic effect of PDT and mTOR inhibition. Without light irradiation, the combinational group still exhibited a slight decrease of viability in 4T1 cells, which may be attributed to the YAP inhibition effect from Verteporfin in the dark environment and its combinational effect with mTOR inhibition (Figure S1, Supporting Information). For MDA‐MB‐231 cells, it showed similar results of synergistic effect upon light irradiation with an average synergy score ≈10 (Figure 2C,D). Taken together, these results indicated that mTOR inhibition from Torin 1 had a strong synergistic effect with Verteporfin‐induced PDT and YAP inhibition in breast cancer cell lines, which held the potential to improve the efficacy of PDT and provide efficient treatment for triple‐negative breast cancer patients.

### 2.2 Preparation and Characterization of VP‐Torin1 NPs

The current administration routes of photosensitizers and mTOR inhibitors in the clinic are intravenous injection or oral administration.^[^ ^22^ ^]^ However, such systemic administration will lead to a series of side effects, such as skin toxicity from photosensitizers and thrombocytopenia from mTOR inhibitors.^[^ ^23^ ^]^ To reduce side effects and increase targeting efficiency, nanoparticle drug delivery systems have been developed, which can improve the solubility and stability of hydrophobic drug molecules and promote passive targeting to tumor tissues through enhanced permeability and retention (EPR) effect.^[^ ^24^ ^]^ Interestingly, we found that Verteporfin and Torin 1 could form carrier‐free nanoparticles without any excipients by the flash nanoprecipitation method (Figure 2E). The size and polydispersity index (PDI) could be influenced by the feeding ratio between Torin 1 and Verteporfin (Figure S2A, Supporting Information). According to their synergy score with light irradiation, we chose the weight ratio of 2.5 to fabricate nanoparticle formulation (VP‐Torin1 NPs). The size, PDI, and Zeta potential of VP‐Torin1 NPs were ≈106 nm, 0.21, and −22 mV, respectively (Figure 2F; Figure S2B, Supporting Information). The transmission electron microscopy (TEM) image showed VP‐Torin1 NPs had spherical morphology with a narrow size distribution (Figure 2G). In addition, VP‐Torin1 NPs could keep stable in phosphate buffer saline (PBS), FBS‐containing DMEM medium, or FBS solution at 37 °C for at least 48 h without obvious aggregation or dissociation, indicating the ability to keep stable during blood circulation with good serum stability (Figure 2H; Figures S2C,S3A, Supporting Information). To test the long‐term stability at storage conditions, VP‐Torin1 NPs were stored in aqueous solutions at 4 °C. The dynamic light scattering (DLS) results showed that the nanoparticles remained stable for at least 16 days, with a particle size ≈110 nm and a PDI ≈0.25 under these storage conditions (Figure S3B, Supporting Information). Then, we used high performance liquid chromatography (HPLC) to determine the drug loading. The encapsulation efficiency of Verteporfin and Torin 1 was 16.25 ± 0.13% and 19.17 ± 0.13%, respectively, and the drug loading capacity of Verteporfin and Torin 1 was 67.94 ± 0.27% and 32.06 ± 0.27%, respectively.

To investigate the self‐assembly mechanism between Verteporfin and Torin 1, we tested the size and PDI values of Torin 1 suspensions, Verteporfin suspensions, or their simple mixtures (Figure S4, Supporting Information). The results showed that all of them could not form well‐dispersed nanoparticles with suitable size (20–200 nm) and PDI value (0–0.25). Afterward, spectroscopy was used to explore the interactions between Verteporfin and Torin 1. VP‐Torin1 NPs exhibited distinct absorption peaks at 254, 420, and 690 nm, indicating both Torin 1 and Verteporfin were successfully encapsulated into the nanoparticles (Figure 2I). Compared with free Verteporfin, VP‐Torin1 NPs showed slightly widen and red‐shifted Soret bands (420 nm) and Q bands (500–750 nm), demonstrating the strong *π–π* stacking interaction between benzoporphyrin structure of Verteporfin and aromatic groups of Torin 1. After adding the surfactant sodium dodecyl sulfate (SDS) that could influence hydrophobic interactions of other molecules, the peak at 420 nm increased remarkably and the peak at 690 nm had an obvious blue‐shift, implying that hydrophobic interactions contributed to the self‐assembly of the two molecules (Figure 2J). However, with increased ion strength, such as NaCl solution, there was no significant change of UV–vis spectrum, suggesting that electrostatic interactions played minor roles for the self‐assembly (Figure 2K). All of the above indicated that *π–π* stacking and hydrophobic interactions were the main driving forces in the self‐assembly process, which was in line with the molecular docking analysis (Figure 2E). Under physiological conditions (pH = 7.4), two drug molecules were gradually released from nanoparticles, and ≈50% Verteporfin and 55% Torin 1 were released within 24 h (Figure S5A, Supporting Information). Under weak acidic conditions (pH = 6.0), ≈50% Verteporfin and 50% Torin 1 were released within 24 h, which was similar to the pH 7.4 condition (Figure S5B, Supporting Information). However, during the first 4 h, the Verteporfin release at pH 6.0 (44%) was slightly slower than that at pH 7.4 (48%). This could be attributed to the fact that the ionization of the carboxyl group in Verteporfin was slightly suppressed under weak acidic conditions, which might influence the interactions between the two drug molecules and their release. Moreover, the PDT effect of VP‐Torin1 NPs was tested by SOSG probe, and the results showed that VP‐Torin1 NPs could generate singlet oxygen (^1^O_2_) upon NIR light irradiation in a time‐dependent manner (Figure S6, Supporting Information). In comparison, the ^1^O_2_ generation ability of VP‐Torin1 NPs was slightly higher than that of free Verteporfin with equivalent concentration, which might be attributed to the fact that VP‐Torin1 NPs dispersed better than free Verteporfin in aqueous solution. To conclude, Verteporfin and Torin 1 could form stable carrier‐free nanoparticles via *π–π* stacking and hydrophobic interactions. With the help of nanoparticle formulation, two drug molecules were expected to be delivered into tumor tissues with high efficiency for synergistic therapy.

### 2.3 Enhanced PDT Effect of VP‐Torin1 NPs

**Figure 3:**
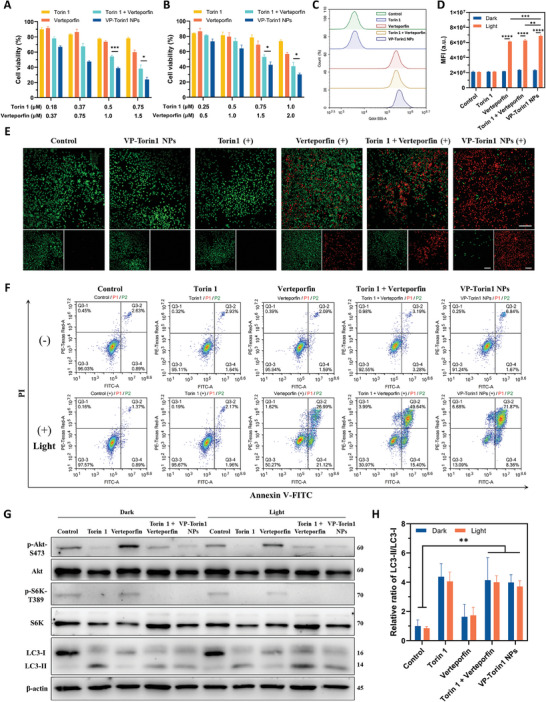
Cytotoxicity and mTOR inhibition effect of VP‐Torin1 NPs. Cell viability of 4T1 cells (A) and MDA‐MB‐231 cells (B) after the treatment of gradient concentrations of Torin 1, Verteporfin, Torin 1 plus Verteporfin, or VP‐Torin1 NPs in the presence of light irradiation (*n* = 3, Xe lamp, 690 nm, 2.7 mW cm^−^^2^, 2 min). C) Flow cytometric analysis of cellular uptake behavior in 4T1 cells treated with Torin 1, Verteporfin, Torin 1 plus Verteporfin, or VP‐Torin1 NPs for 4 h. D) ROS generation level of 4T1 cells after treatment with Torin 1, Verteporfin, Torin 1 plus Verteporfin, or VP‐Torin1 NPs with or without light irradiation (*n* = 3, Xe lamp, 690 nm, 2.7 mW cm^−^^2^, 10 min). DCFH‐DA was used as the indicator. E) Representative confocal microscopy images of Calcein AM (green fluorescence, indicating living cells) and PI (red fluorescence, indicating dead cells) staining in 4T1 cells after treatment with Torin 1, Verteporfin, Torin 1 plus Verteporfin, or VP‐Torin1 NPs with or without light irradiation (Xe lamp, 690 nm, 2.7 mW cm^−^^2^, 2 min, Scale bar: 200 µm). F) Flow cytometric apoptosis analysis of 4T1 cells treated with Torin 1, Verteporfin, Torin 1 plus Verteporfin, or VP‐Torin1 NPs with or without light irradiation (Xe lamp, 690 nm, 2.7 mW cm^−^^2^, 2 min). G) Western blot analysis of mTOR downstream protein levels (p‐Akt, Akt, p‐S6K, S6K, and LC3‐I/II) in 4T1 cells receiving indicated treatments with or without light irradiation (Xe lamp, 690 nm, 2.7 mW cm^−^^2^, 90 s). H) Quantitative Western Blot analysis of LC3‐II / LC3‐I. Data were shown as mean ± SD. “(+)” represents the application of light irradiation. *p < 0.05, **p < 0.01, ***p < 0.001, ****p < 0.0001.

In order to evaluate the difference in anti‐cancer effects between free drug molecules and VP‐Torin1 NPs, an MTT assay was conducted on 4T1 cells and MDA‐MB‐231 cells (**Figure** **3**A,B). For 4T1 cells upon light irradiation, Verteporfin plus Torin 1 group showed higher cytotoxicity compared with free Verteporfin or Torin 1 groups, which was consistent with the synergistic studies. Moreover, VP‐Torin1 NPs showed much higher cytotoxicity compared with Verteporfin plus Torin 1 group at gradient concentrations. In particular, Verteporfin (1 µm) plus Torin 1 (0.5 µm) exhibited 54% cell viability, while VP‐Torin1 NPs at the equivalent concentration showed a considerable decrease to 38%. For MDA‐MB‐231 cells, VP‐Torin1 NPs exhibited similar results with enhanced PDT and better anti‐cancer performance. Without light irradiation, VP‐Torin1 NPs also showed slightly higher cytotoxicity (Figure S7, Supporting Information). Live/dead staining assay demonstrated consistent results that almost all 4T1 cells incubated with VP‐Torin1 NPs were dead with strong red fluorescence upon light irradiation (Figure 3E). Apoptosis assay further verified the increased cytotoxicity of VP‐Torin1 NPs plus light irradiation, with 77% 4T1 cells in early and late apoptosis stages (Figure 3F; Figure S8, Supporting Information). Particularly, the late apoptosis ratio of VP‐Torin1 NPs was ≈1.5 times that of Verteporfin plus Torin 1 group.

To explore the mechanism of enhanced PDT effect by VP‐Torin1 NPs, cellular uptake experiments were conducted in 4T1 cells (Figure 3C; Figures S9,S10, Supporting Information). After 1 and 4  h incubation, VP‐Torin1 NPs exhibited 1.27‐fold and 1.38‐fold cell internalization levels, respectively, compared with Verteporfin plus Torin 1 group, possibly owing to different cellular endocytosis pathways. The intracellular ROS generation level was measured with the help of a 2′,7′‐dichlorofluorescein diacetate (DCFH‐DA) probe (Figure 3D). It was observed that the ROS level of VP‐Torin1 NPs was significantly higher than that of free Verteporfin or Verteporfin plus Torin 1 groups (*p* < 0.01), which could be attributed to the higher internalization level of VP‐Torin1 NPs. The higher cellular uptake and ROS generation levels in contrast to free drug groups might contribute to the enhanced PDT effect of VP‐Torin1 NPs. Besides, confocal laser scanning microscopy (CLSM) imaging assay showed that ROS were generated both in the nucleus and cytoplasm after nanoparticle treatment plus light irradiation (Figure S11, Supporting Information). Since intracellular ROS generation had been reported to promote lysosome escape, we also investigated whether VP‐Torin1 NPs could escape from lysosomes by confocal imaging assay (Figure S12, Supporting Information).^[^ ^25^ ^]^ The results showed that VP‐Torin1 NPs had high colocalization with lysosomes after 1 h incubation in both groups. After light irradiation at a 1 h time point, cells were incubated for another 3 h. The 4 h images showed obvious lysosome escape of VP‐Torin1 NPs upon light irradiation with decreased colocalization proportion, while the dark group still had high colocalization with lysosomes after 4 h incubation.

After nanoparticle endocytosis and lysosome escape, Torin 1 could be released in the cytoplasm and inhibit mTORC1/C2 downstream signaling pathways in cancer cells. As mTORC1 substrates, S6 kinases (S6K) were involved in the regulation of protein translation and anabolism. For mTORC2, it could phosphorylate and activate downstream Akt proteins to regulate cell proliferation and migration.^[^ ^26^ ^]^ The western blot results showed that VP‐Torin1 NPs could notably inhibit the phosphorylation of S6K and Akt proteins, mainly from the mTORC1/C2 dual inhibition effect of Torin 1 (Figure 3G). Apart from the phosphorylation, mTOR also played an important role in autophagy regulation. It had been reported that mTOR inhibition could enhance autophagy levels and promote protein or organelle degradation, leading to autophagic cell death.^[^ ^27^ ^]^ During autophagy, LC3‐I would become LC3‐II after a ubiquitin‐like enzymatic reaction and translocate from the cytoplasm to the membrane of autophagosomes. The western blot result demonstrated that VP‐Torin1 NPs could improve the LC3‐II/LC3‐I ratio compared with control groups, suggesting the formation of autophagosomes and enhanced autophagy level to promote anti‐cancer effect (Figure 3H). Taken together, VP‐Torin1 NPs owned higher cellular uptake, ROS generation, and apoptosis levels compared with free drug molecules. Besides, VP‐Torin1 NPs could significantly downregulate mTORC1/C2 downstream signaling pathways and promote autophagic flux for enhanced PDT in breast cancer cell lines.

### 2.4 Combinational Anti‐Angiogenesis Effect of VP‐Torin1 NPs

Due to the abnormal glycolysis in solid tumor tissues, the tumor microenvironment is acidic and lacks of nutrients and oxygen, which can result in genetic and epigenetic alterations in cancer and immune cells to promote tumor progression.^[^ ^28^ ^]^ However, during PDT, the photosensitizer Verteporfin needs to consume oxygen to generate ROS upon light irradiation, which may exacerbate local hypoxia. The PDT‐evoked tumor hypoxia will further lead to pathological angiogenesis and cancer metastasis. To evaluate the anti‐angiogenesis effect of VP‐Torin1 NPs, wound healing assay was conducted in human umbilical vein endothelial cells (HUVECs) (Figure S14, Supporting Information). The results showed that both Torin 1 and Verteporfin could inhibit the lateral migration of HUVECs with 62% and 25% inhibition rate, respectively, at 24 h without light irradiation, maybe due to mTOR and YAP inhibition effects. For the VP‐Torin1 NPs group, it had a 1.17‐fold inhibition level compared with Verteporfin plus Torin 1 at 24 h. Since tumor tissue is surrounded by blood vessels for nutrient supply, the nanoparticles have the chance to interact with newborn endothelial cells to exert an anti‐angiogenesis effect after retention at tumor tissues.

**Figure 4:**
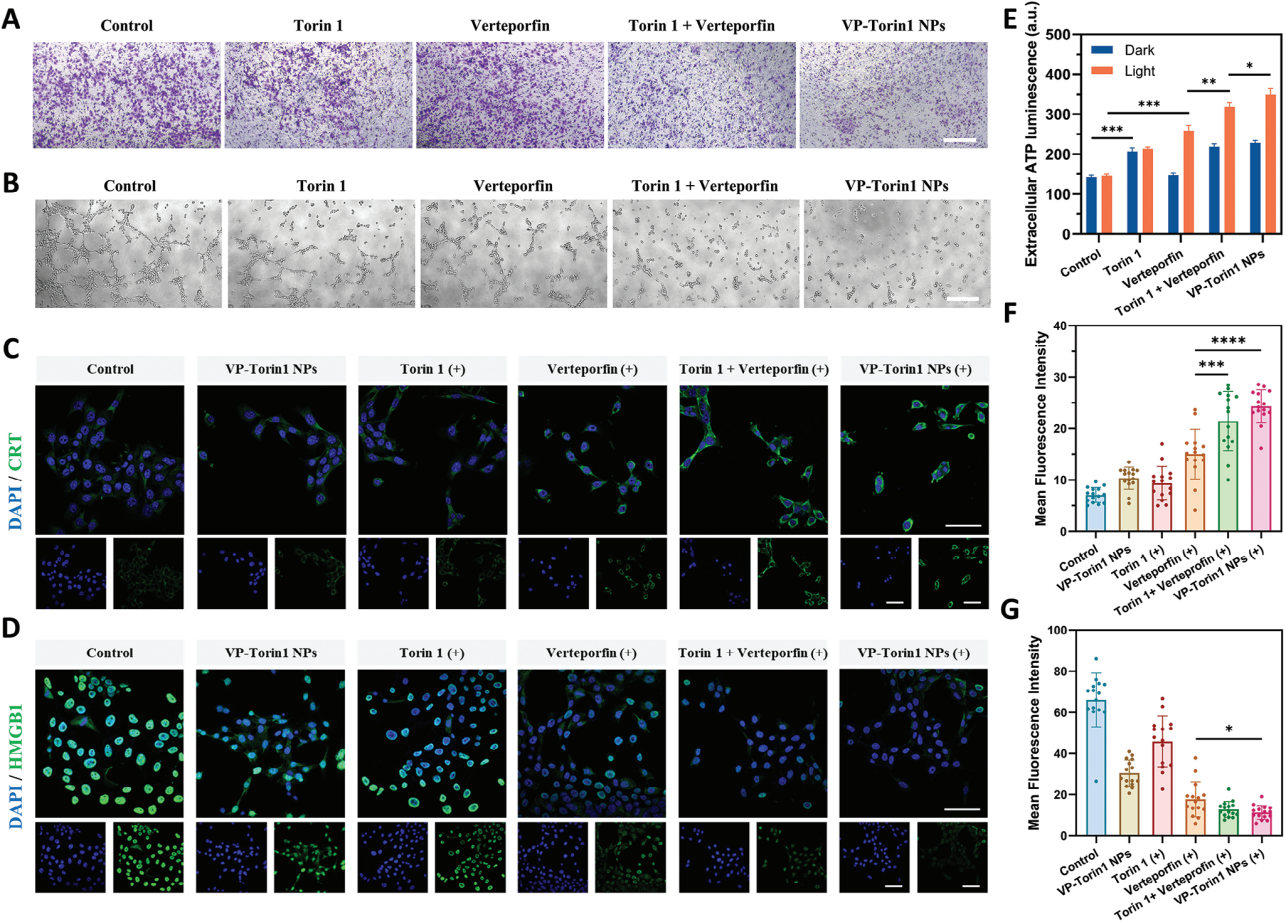
Anti‐angiogenesis and immunogenic cell death (ICD) induced by VP‐Torin1 NPs. A) Migration ability of HUVECs treated with Torin 1, Verteporfin, Torin 1 plus Verteporfin, or VP‐Torin1 NPs (Scale bar: 0.5 mm). B) Tube formation ability of HUVECs treated with Torin 1, Verteporfin, Torin 1 plus Verteporfin, or VP‐Torin1 NPs (Scale bar: 0.5 mm). C) Representative confocal microscopy images of CRT proteins in 4T1 cells treated with indicated formulations (CRT protein in green and nucleus in blue, Xe lamp, 690 nm, 2.7 mW cm^−^^2^, 90 s, Scale bar: 50 µm). D) Representative confocal microscopy images of HMGB1 proteins in 4T1 cells treated with indicated formulations (HMGB1 protein in green and nucleus in blue, Xe lamp, 690 nm, 2.7 mW cm^−^^2^, 90 s, Scale bar: 50 µm). E) Extracellular ATP levels of 4T1 cells treated with indicated formulations. ATP concentrations were represented by luminescence intensity (Xe lamp, 690 nm, 2.7 mW cm^−^^2^, 90 s). F) Quantified mean fluorescence intensity of CRT in 4T1 cells receiving different formulations. G) Quantified mean fluorescence intensity of HMGB1 in 4T1 cells receiving different formulations. Data were shown as mean ± SD. “(+)” represents the application of light irradiation. *p < 0.05, **p < 0.01, ***p < 0.001, ****p < 0.0001.

In addition, a vertical migration assay was conducted with the help of transwell inserts (**Figure** **4**A). It was observed that both Torin 1 and Verteporfin could inhibit vertical migration and Verteporfin plus Torin 1 exhibited a combination effect with only an 18.3% migration rate without light irradiation (Figure S15, Supporting Information). Similarly, VP‐Torin1 NPs showed a lower migration rate and better anti‐angiogenesis effect. In tube formation assay, HUVECs would form tubes, branches, or node structures after seeded on the Matrigel in normal conditions (Figure 4B). However, the formation of tubes was significantly restrained by free Torin 1 and Verteporfin, and VP‐Torin1 NPs showed a better inhibition effect. To explore the mechanism of anti‐angiogenesis, western blot analysis demonstrated that VP‐Torin1 NPs could effectively inhibit the phosphorylation of Akt and S6K that were related to proliferation and migration in HUVECs (Figure S16A, Supporting Information). Furthermore, VP‐Torin1 NPs could significantly reduce the mRNA expression level of CTGF, Cyr61, EGFR, and AREG in HUVECs without light irradiation, all of which were YAP downstream signaling pathways and associated with hypoxia‐elicited angiogenesis (Figure S16B, Supporting Information). These data suggested that VP‐Torin1 NPs had a combinational anti‐angiogenesis effect, mainly from the mTORC1/C2 dual inhibition from Torin 1 and YAP inhibition from Verteporfin. mTOR and YAP inhibition from two drug molecules had distinct mechanisms of action for anti‐angiogenesis, which presented combinational effect in anti‐angiogenesis. Such therapeutic function was expected to reshape the tumor microenvironment and reverse the vascular angiogenesis and tumoral aggressiveness from PDT‐evoked hypoxia.

### 2.5 In Vitro ICD Effect

Apart from ROS generation, it was reported that PDT could induce ICD effect and promote the release of DAMPs, such as ATP, calreticulin (CRT), and high mobility group box 1 (HMGB1), from dying cancer cells to activate DC maturation and T cell cytotoxicity.^[^ ^29^ ^]^ First, the extracellular ATP secretion level of 4T1 cells after different treatments was measured by a luminescence kit. The result showed that Torin 1 and Verteporfin (light) had 1.45‐fold and 1.81‐fold secretion levels, respectively, compared with control groups (Figure 4E). Verteporfin plus Torin 1 group exhibited a combinational effect in contrast to mono drug groups and VP‐Torin1 NPs owned slightly higher levels of ATP than that of free drug groups under light irradiation. The reasons for the Torin 1‐elicited ICD effect might be mTOR inhibition and autophagic flux induction, which was consistent with previous studies that autophagy could boost ATP secretion and the ICD process to activate purinergic receptors and anti‐cancer immunity.^[^ ^30^ ^]^

The other DAMPs were CRT and HMGB1. The CLSM images showed that there was quite a low CRT expression level on the 4T1 cell surface under normal conditions (Figure 4C). However, VP‐Torin1 NPs treatment could significantly upregulate CRT expression level upon light irradiation, which could serve as an “eat me” signal to promote antigen presentation (Figure 4F). Regarding HMGB1, the results demonstrated that there were abundant HMGB1 proteins in the nuclei in the control group (Figure 4D). After VP‐Torin1 NPs treatment, there was a dramatic decrease of HMGB1 from nuclei compared with other free drug groups upon light irradiation, which could function as a “danger signal” and recruit immune cells (Figure 4G). These results suggested that Verteporfin and Torin 1 had the combinational effect to activate the anti‐cancer immune response and VP‐Torin1 NPs owned a much stronger ICD effect compared with free drug molecules. One of the reasons for poor prognosis in triple‐negative breast cancer patients is the high metastasis rate. Therefore, the anti‐cancer immunity triggered by VP‐Torin1 NPs has the potential to reverse tumor immunosuppressive microenvironment and prevent metastasis in clinical practice.

### 2.6 Tumor Accumulation and Antitumor Effect in 4T1 Tumor Model

**Figure 5:**
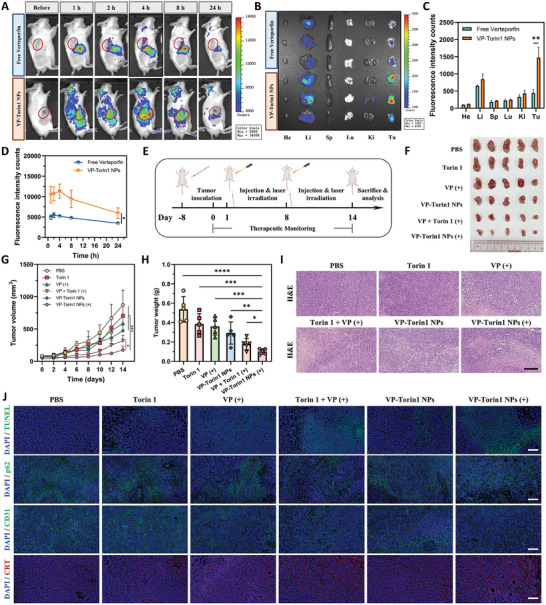
Biodistribution and anti‐cancer effect of VP‐Torin1 NPs in 4T1 tumor‐bearing BALB/c mouse model. A) Fluorescence imaging and biodistribution of Verteporfin and VP‐Torin1 NPs at different time intervals after intravenous injection in vivo. The tumors are indicated by red circles. B) Ex vivo fluorescence imaging of major organs and tumor tissues examined at 24 h post‐injection. C) Quantitative fluorescence analysis of major organs and tumor tissues examined at 24 h post‐injection (*n* = 3). D) Quantitative fluorescence analysis of Verteporfin and VP‐Torin1 NPs in tumor tissues at different time intervals after intravenous injection. E) Treatment schedule of combinational therapy by VP‐Torin1 NPs for improved PDT and tumor microenvironment remodeling in 4T1 tumor‐bearing BALB/c mouse model. F) The image of tumors collected from tumor‐bearing mice with different treatments at the end of the antitumor study ex vivo (*n* = 5). G) Tumor volume growth profiles of tumor‐bearing mice with different treatments during the 14‐day therapeutic period (*n* = 5). H) The weight of tumors from tumor‐bearing mice with different treatments at the end of the antitumor study (*n* = 5). I) Hematoxylin and eosin (H&E) staining of tumor tissues in different treatment groups (Scale bar: 200 µm). J) Representative confocal microscopy images of TUNEL assay, p62, CD31, and CRT proteins in the tumor tissues of different treatment groups (Scale bar: 100 µm). Data were shown as mean ± SD. “(+)” represents the application of light irradiation. *p < 0.05, **p < 0.01, ***p < 0.001, ****p < 0.0001.

For VP‐Torin1 NPs, they were anticipated to circulate in the blood vessels after intravenous administration and gradually accumulate in tumor tissues based on passive targeting. To test this hypothesis, 4T1 tumor‐bearing BALB/c mouse model was established. After intravenous injection, VP‐Torin1 NPs showed much higher fluorescence intensity in tumor tissues compared with free Verteporfin at an equivalent concentration (**Figure** **5**A,D). At the 4 h time point, the VP‐Torin1 NPs group reached a maximum concentration that was ≈2.17 times that of the free Verteporfin group. At 24 h after administration, main organs and tumor tissues were collected to detect ex vivo fluorescence. In the VP‐Torin1 NPs group, nanoparticles mainly accumulated in liver and tumor tissues, which was consistent with previous reports (Figure 5B,C).^[^ ^31^ ^]^ Regarding average fluorescence intensity, tumors had 1.75‐fold intensity counts compared with livers, demonstrating the superior passive targeting property. In line with whole‐body imaging, the fluorescence of VP‐Torin1 NPs in tumors was 3.3 times that of the free Verteporfin group. These results indicated that VP‐Torin1 NPs owned prolonged blood circulation and better tumor accumulation ability, which could reduce side effects and improve biosafety after administration.

To investigate the in vivo anti‐cancer effect of VP‐Torin1 NPs, 4T1 tumor‐bearing BALB/c mice were randomly divided into 6 groups with different treatments for 2 weeks (Figure 5E). On day 14, tumor tissues were harvested, photographed, and weighed. The results showed that Verteporfin plus Torin 1 group under light irradiation exhibited better tumor growth inhibition performance in contrast to Torin 1 or Verteporfin under light irradiation alone, suggesting the in vivo synergistic effect of two drug molecules under light irradiation (Figure S17, Supporting Information). Importantly, the VP‐Torin1 NPs group under light irradiation owned the highest antitumor efficacy, compared with Verteporfin plus Torin 1 (*p* < 0.05) and other groups (*p* < 0.001) (Figure 5F,G). As for tumor weight, the VP‐Torin1 NPs group was 56% of the Verteporfin plus Torin 1 group, possibly due to better tumor targeting properties of VP‐Torin1 NPs. Consistently, the tumor inhibition rate in the nanoparticles group was 19%, which is significantly lower than 34% for the Verteporfin plus Torin 1 group (Figure S18, Supporting Information). In addition, hematoxylin and eosin (H&E) and terminal deoxynucleotidyl transferase dUTP nick end labeling (TUNEL) staining assays of tumor tissue sections also demonstrated a higher proportion of necrosis and apoptosis in the VP‐Torin1 NPs treated group with light irradiation in contrast to other groups (Figure 5I,J).

During the 14‐day treatment, there was no significant difference on body weight, alanine aminotransferase (ALT), and aspartate transaminase (AST) assays among the 6 groups (Figure S19, Supporting Information). Moreover, H&E staining of major organs (heart, liver, spleen, lung, and kidney) showed that there was no histological and morphological change after the treatment with different formulations (Figure S20, Supporting Information). The photographs of the 4T1 tumor‐bearing mice during treatment show no evident skin burns or toxicity after various treatments (Figure S21, Supporting Information). To conclude, these data suggest that VP‐Torin1 NPs with light irradiation could efficiently inhibit tumor growth in mouse models without systemic toxicity and severe side effects.

### 2.7 Tumor Microenvironment Remodeling and Antitumor Immune Response

Since Torin 1 could inhibit angiogenesis and promote the PDT‐elicited ICD process by mTORC1/C2 dual inhibition and autophagy induction, we supposed that the combination of Verteporfin and Torin 1 might contribute to reshape the aggressive and immunosuppressive tumor microenvironment in vivo by anti‐angiogenesis and photoimmunotherapy. The immunofluorescence assay of tumor sections showed that VP‐Torin1 NPs with light irradiation could significantly downregulate the expression levels of p62 and CD31 and upregulate CRT expression level compared with free drug groups (Figure 5J). The p62 expression level is inversely correlated with autophagic activity, and the CD31 (as known as PECAM‐1) marker represents the presence of endothelial cells and the degree of tumor angiogenesis. Therefore, these data indicate that VP‐Torin1 NPs could efficiently deliver Torin 1 into tumor tissues, promote autophagic cell death, and inhibit angiogenesis in the microenvironment.

**Figure 6:**
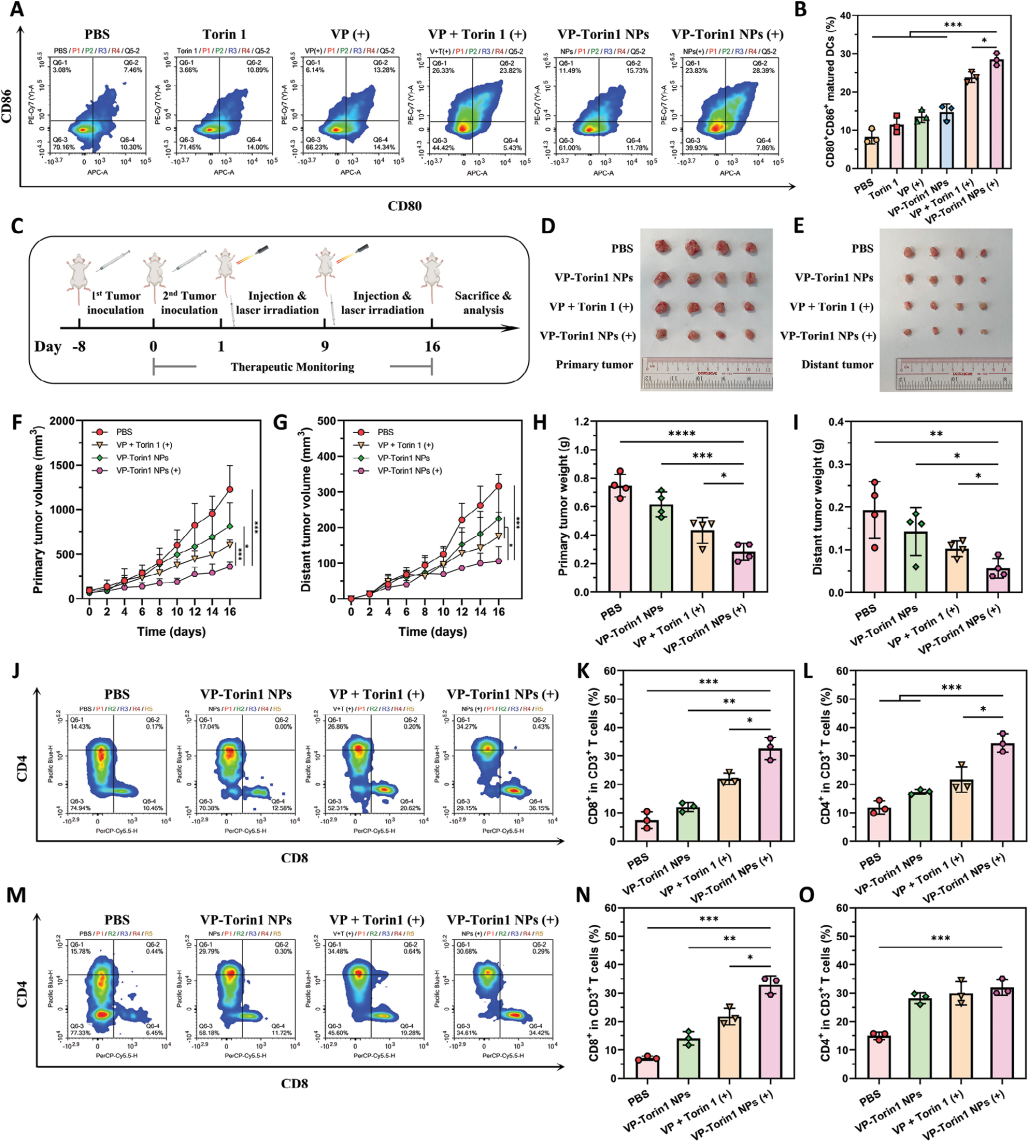
Tumor microenvironment remodeling and anti‐cancer immune effect of VP‐Torin1 NPs. A) Flow cytometric analysis of DC maturation in tumor‐draining lymph nodes collected from 4T1 tumor‐bearing mice with different treatments at the end of the antitumor study. B) Quantitative analysis of DC maturation in the tumor‐draining lymph nodes (*n* = 3). C) Schematic illustration of anti‐cancer immunity studies in bilateral 4T1 tumor‐bearing BALB/c mouse model. The image of primary tumors (D) and distant tumors (E) collected from bilateral 4T1 tumor‐bearing mice with different treatments at the end of the immunotherapy study ex vivo (*n* = 4). Primary tumor (F) and distant tumor (G) volume growth profiles of the mice with different treatments during the 16‐day therapeutic period (*n* = 4). The weight of primary tumors (H) and distant tumors (I) at the end of the immunotherapy study (*n* = 4). J) Flow cytometric analysis of infiltrating T cells in primary tumor tissues collected from bilateral 4T1 tumor‐bearing mice with different treatments at the end of the study. K) Quantitative analysis of CD8^+^ T cells in primary tumor tissues (*n* = 3). L) Quantitative analysis of CD4^+^ T cells in primary tumor tissues (*n* = 3). M) Flow cytometric analysis of infiltrating T cells in distant tumor tissues collected from bilateral 4T1 tumor‐bearing mice with different treatments at the end of the study. N) Quantitative analysis of CD8^+^ T cells in distant tumor tissues (*n* = 3). O) Quantitative analysis of CD4^+^ T cells in distant tumor tissues (*n* = 3). Data were shown as mean ± SD. “(+)” represents the application of light irradiation. *p < 0.05, **p < 0.01, ***p < 0.001, ****p < 0.0001.

Apart from PDT‐evoked hypoxia and angiogenesis, the other limitation of current therapies is immunosuppression, referred as “immunologically cold tumor”. CRT staining showed that the VP‐Torin1 NPs group resulted in a higher expression level in contrast to control groups, demonstrating the synergistic ICD effect (Figure 5J). Given that DAMPs release could facilitate antigen presentation and DC maturation, tumor‐draining lymph nodes (TDLNs) from 4T1 tumor‐bearing mice were collected after the endpoint. Flow cytometry analysis showed that the percentage of matured DCs (CD80^+^CD86^+^) in CD45^+^MHCII^+^CD11c^+^ immune cells in VP‐Torin1 NPs groups upon light irradiation was significantly higher than that of Verteporfin plus Torin 1 (*p* < 0.05) and other groups (*p* < 0.001), implying considerable DC maturation and strong immune response after nanoparticle treatment (**Figure** **6**A,B).

The aforementioned results demonstrated that VP‐Torin1 could remodel the tumor microenvironment by anti‐angiogenesis, DAMPs release, and DC maturation. Since tumor metastasis was quite common in triple‐negative breast cancer patients and had become one of the reasons for tumor recurrence and treatment failure, a bilateral syngeneic 4T1 tumor‐bearing mouse model was established to explore the anti‐metastasis effect. Mice were subcutaneously injected with 1 × 10^6^ 4T1 cells into the right flank to simulate primary tumors (Figure 6C). Eight days later, 8 × 10^5^ 4T1 cells were subcutaneously injected into the left flank as distant tumors. Since the syngeneic distant tumor was on the opposite side and smaller in size, it could simulate a metastatic tumor.^[^ ^13^, ^32^ ^]^ After intravenous injection of different formulations, NIR light irradiation was only given to the primary tumor at the right side. In this way, distant tumors were supposed to be eliminated only by systemic immunity to evaluate antitumor immune response. The tumor volume curve of primary tumors showed that VP‐Torin1 NPs with light irradiation could successfully delay the tumor growth with smaller tumor size and lower weight compared with other groups, which was consistent with the above results (Figure 6D,F,H). Regarding distant tumors, it was observed that VP‐Torin1 NPs with light irradiation could also effectively inhibit distant tumor growth during 16‐day treatment (Figure 6E,G). For tumor weight, the VP‐Torin1 NPs group upon light irradiation was 54% of the Verteporfin plus Torin 1 group, suggesting strong distant tumor suppression and abscopal effect (Figure 6I).

To investigate the mechanism of distant therapeutic effect, T cell infiltration in tumors was analyzed by flow cytometry. In primary tumors, the proportion of cytotoxic CD8^+^ or helper CD4^+^ in tumor‐infiltrating CD3^+^ T cells in the VP‐Torin1 NPs group with light irradiation was ≈1.48 or 1.59 times that of Verteporfin plus Torin 1 group. This may be due to a stronger ICD effect and a higher level of DC maturation (Figure 6J–L). Likewise, in distant tumors, VP‐Torin1 NPs had significantly higher infiltration of cytotoxic CD8^+^ T cells compared with Verteporfin plus Torin 1 (*p *< 0.05) and other groups (*p* < 0.01), which could explain the augmented abscopal effect in distant tumors (Figure 6M–O). However, there was no significant difference in tumor‐infiltrating helper CD4^+^ T cells among the treatment groups, possibly due to the fact that CD4^+^ T cells had both inflammatory cytotoxic and immunosuppressive regulatory effects for metastatic cancer cells.^[^ ^33^ ^]^ Additionally, systemic toxicity was not observed, and there was no significant difference in body weight among the four groups of bilateral 4T1 tumor‐bearing mice (Figure S22, Supporting Information). Collectively, these data suggested that VP‐Torin1 NPs could effectively elevate systemic anti‐cancer immune response, promote the infiltration of cytotoxic CD8^+^ T cells, and inhibit distant tumor growth by co‐delivering two drugs through nanoparticle formulation, which held the potential to restrain cancer cell metastasis and improve the prognosis for triple‐negative breast cancer patients.

The designed VP‐Torin1 NPs were composed of two small drug molecules with good stability and could integrate mTOR inhibition with photodynamic therapy for tumor microenvironment remodeling to provide safe and efficient breast cancer treatment. Such carrier‐free nanodrug systems could improve drug efficacy, avoid possible side effects related to carrier materials, and facilitate large‐scale production with simple formulation to provide long‐term benefits. However, there are still some challenges in clinical translation. The nanodrug systems mainly rely on passive targeting to accumulate at tumor tissues, so the accumulation might be insufficient and vary among individuals.^[^ ^19^, ^20^ ^]^ Besides, the proportion of two drug molecules within nanoparticles during industrial production needs to be strictly controlled to ensure the synergistic effect. Last, the clinical settings of external light sources need to be adjusted from animal studies to human patients.

## 3 Conclusion

In this study, our findings provide solid evidence for the synergistic effect between the mTORC1/C2 dual inhibitor Torin 1 and the photosensitizer Verteporfin in improving PDT and remodeling the tumor microenvironment. For targeting delivery, we developed a simple and stable carrier‐free nanodrug formulation self‐assembled by Verteporfin and Torin 1 with the help of *π–π* stacking and hydrophobic interactions. The nanoparticle system demonstrates enhanced anti‐cancer efficacy, combinational anti‐angiogenesis, and stronger ICD effect upon NIR light irradiation. In vivo results indicate that the nanoparticles can efficiently accumulate at tumor tissues, suppress tumor growth, and reshape immunosuppressive tumor microenvironment through mTOR inhibition‐enhanced PDT, angiogenesis inhibition, and anti‐cancer immune activation. For metastatic cancer cells, our system also exhibits considerable inhibition effect on distant tumor growth through activated cytotoxic T cells and systemic antitumor immune response with high biocompatibility and low side effects. Therefore, our carrier‐free nanodrug system holds great potential to provide safe and efficient treatment for triple‐negative breast cancer patients in clinical applications in the near future.

## 4 Experimental Section

### Materials

Photosensitizer Verteporfin (VP), crystal violet, and DNase I were purchased from Macklin (Shanghai, China). mTOR inhibitor Torin 1 and 2′,7′‐Dichlorodihydrofluorescein diacetate (DCFH‐DA) were obtained from MedChemExpress (MCE, Shanghai, China). Dimethyl sulfoxide (DMSO) was obtained from Sigma‐Aldrich (Darmstadt, Germany). Oxygen Sensor Green (SOSG) assay, Hoechst 33342, Lysotracker, DAPI, and Calcein AM were obtained from Thermo Fisher (MA, USA). 2,5‐diphenyl‐2‐H‐tetrazolium bromide (MTT) was purchased from J&K Chemical (Beijing, China). Apoptosis kit, TUNEL assay kit, and propidium iodide (PI) were purchased from Beyotime (Shanghai, China). A luminescent ATP measurement kit was ordered from Abcam (Cambridge, UK). Matrigel was obtained from BD Bioscience (New Jersey, US). Acetonitrile (ACN), methanol (MeOH), and other solvents were purchased from Oriental Co., Ltd (Hong Kong, China). Antibodies used in this project are listed in Table S1 (Supporting Information).

### Cell Culture

The human triple‐negative breast cancer cell line (MDA‐MB‐231), human umbilical vein endothelial cell (HUVEC), and mouse breast cancer cell line (4T1) were purchased from ATCC. Cells were cultured in DMEM (Gibco) supplemented with 10% FBS (Gibco) and 1% penicillin/streptomycin (Gibco) at 37 °C with 5% CO_2_.

### Preparation and Characterization of Verteporfin‐Torin 1 Nanoparticles (VP‐Torin1 NPs)

The Verteporfin (3.75 mg mL^−1^) and Torin 1 (1.5 mg mL^−1^) were dissolved in DMSO, respectively. Afterward, 3 µL Verteporfin and 3 µL Torin 1 were mixed and vortexed thoroughly. The drug mixture solution was quickly added into 600 µL ddH_2_O using the nanoprecipitation method and stirred for 5 min to form VP‐Torin1 NPs. The nanoparticles were further purified by centrifugation (20 000 g, 30 min) to remove the organic solvent and unencapsulated drug molecules. The nanoparticles were then sonicated and centrifuged at 3000 g for 5 min to remove large aggregates. The size and polydispersity index (PDI) were measured by DLS (Malvern Zetasizer). TEM images were captured by FEI Tecnai G2 20 Scanning transmission electron microscope. The nanoparticles were also dissolved in PBS, FBS‐containing DMEM medium, or FBS solution for stability test (37 °C, 48 h). To test the long‐term stability, the nanoparticles were dispersed in water and stored at 4 °C. At days 0, 3, 6, 9, 13, and 16, the size and PDI were measured by DLS. The UV–vis absorption spectra of different formulations in H_2_O or methanol were measured by SpectraMax M4 (Molecular Devices LLC, San Jose, CA). The concentration of loaded drug molecules was determined by HPLC. Encapsulation efficiency and loading capacity were calculated as follows:

(1) $$\mathrm{Encapsulation}\;\mathrm{efficiency}\;\;\left(\%\right)=\frac{\mathrm{Weight}\;\mathrm{of}\;\mathrm{loaded}\;\mathrm{drug}}{\mathrm{Weight}\;\mathrm{of}\;\mathrm{feeded}\;\mathrm{drug}}\;\times 100\%$$

(2) $$\mathrm{Loading}\;\mathrm{capacity}\;\;\left(\%\right)=\frac{\mathrm{Weight}\;\mathrm{of}\;\mathrm{loaded}\;\mathrm{drug}}{\mathrm{Weight}\;\mathrm{of}\;\mathrm{nanoparticles}}\times 100\%$$

### Drug Release

The release profile of Torin 1 and Verteporfin from VP‐Torin1 NPs was assessed using the dialysis method. VP‐Torin1 NPs (600 µL) were placed in the dialysis bag (3500 Da MWCO) and dialyzed against 7 mL of PBS buffer (pH = 7.4) at 37 °C. At pre‐determined time points (0.5, 1, 2, 4, 6, 8, and 24 h), the entire external PBS solution was replaced with a fresh PBS solution. To explore the drug release profile at pH 6.0, 600 µL of VP‐Torin1 NPs were placed in a dialysis bag (3500 Da MWCO) and dialyzed against 7 mL PBS solution (pH = 6.0) at 37 °C. At pre‐determined time points (0.5, 1, 2, 4, 6, 8, and 24 h), the entire external solution was replaced with fresh PBS solution (pH = 6.0). The cumulative release percentage of Torin 1 and Verteporfin over time was determined by HPLC analysis.

### Singlet Oxygen (^1^O_2_) Detection

VP‐Torin1 NPs or equivalent Verteporfin were mixed with SOSG (1 µm) in 600 µL H_2_O. In the presence of light irradiation (Laser, 690 nm, 50 mW cm^−^^2^), the fluorescence intensity at 530 nm and fluorescent spectrum from 510 to 600 nm were determined by SpectraMax M4 (Interval: 30 s, Duration: 5 min, Excitation wavelength: 488 nm).

### Cytotoxicity Assay

4T1 cells (8 × 10^3^) or MDA‐MB‐231 cells were seeded in 96‐well plates and cultured overnight. Cells were incubated with gradient concentrations of Verteporfin, Torin 1, or dual‐drug combination for 24 h. Then the cells were washed with PBS and cultured in fresh media with or without light irradiation (Xe lamp, 690 nm, 2.7 mW cm^−^^2^, 1 min). After 24 h, 10 µL MTT solution (5 mg mL^−1^) was added into each well and incubated with cells for 4 h. Then, the media were replaced by 100 µL DMSO and the absorbance at 490, 570, and 630 nm was measured by SpectraMax M4 to calculate cell viability (normalized to the control group). The synergy scores of Verteporfin and Torin 1 in 4T1 or MDA‐MB‐231 cells were calculated by the HSA model in the Synergy Finder website according to the cell viability data of the MTT assay. To compare the anti‐cancer effect of VP‐Torin1 NPs and free drugs, the cells were incubated with different formulations for 4 h. Then the cells were washed with PBS and cultured in fresh media with or without light irradiation (Xe lamp, 690 nm, 2.7 mW cm^−^^2^, 2 min). After 24 h, the cell viability of each group was determined by MTT assay (normalized to the control group).

### Cellular Uptake

4T1 cells (5 × 10^4^) were seeded in 24‐well plates and cultured overnight. The cells were treated with DMSO, Torin 1, Verteporfin, Torin 1 plus Verteporfin or VP‐Torin1 NPs for 1 or 4 h. Then the cells were washed with PBS three times, trypsinized, and collected. The fluorescence intensity (Qdot 655 channel) was determined by flow cytometry (Agilent NovoCyte Quanteon).

### Live/Dead Cell Staining

4T1 cells (2 × 10^5^) were seeded in confocal dishes (SPL100350) and cultured overnight. The cells were treated with DMSO, Torin 1, Verteporfin, Torin 1 plus Verteporfin or VP‐Torin1 NPs for 4 h. Then the cells were washed with PBS and cultured in fresh media with or without light irradiation (Xe lamp, 690 nm, 2.7 mW cm^−^^2^, 2 min). After 24 h, the cells were incubated with Calcein AM and PI in HEPES buffer for 30 min at 37 °C. The fluorescence was observed by CLSM (Carl Zeiss LSM880).

### Cell Apoptosis

4T1 cells (10^5^) were seeded in 12‐well plates and cultured overnight. The cells were treated with DMSO, Torin 1, Verteporfin, Torin 1 plus Verteporfin or VP‐Torin1 NPs for 4 h. Then the cells were washed with PBS and cultured in fresh media with or without light irradiation (Xe lamp, 690 nm, 2.7 mW cm^−^^2^, 2 min). After 24 h, the cells were washed with PBS three times, trypsinized and harvested, and stained with Annexin‐V/PI apoptosis kit according to the instructions. The apoptosis ratio was analyzed by flow cytometry (Agilent NovoCyte Quanteon).

### ROS Generation

4T1 cells (8 × 10^4^) were seeded in 24‐well plates and cultured overnight. The cells were treated with DMSO, Torin 1, Verteporfin, Torin 1 plus Verteporfin or VP‐Torin1 NPs for 4 h. Subsequently, the cells were washed with PBS three times, cultured in fresh media with 10 µm DCFH‐DA probe, and irradiated with Xe lamp (690 nm, 2.7 mW cm^−^^2^, 10 min). After 30 min incubation in the shield from light, cells were washed with PBS and harvested for flow cytometry analysis (Agilent NovoCyte Quanteon, FITC channel). For confocal imaging, 2.5 × 10^5^ 4T1 cells were seeded in confocal dishes (SPL100350) and cultured overnight. The cells were treated with different formulations for 4 h. Then, the cells were washed with PBS, stained with a DCFH‐DA probe, and irradiated with an Xe lamp (690 nm, 2.7 mW cm^−^^2^, 10 min). Afterward, cells were washed with PBS and incubated with Hoechst 33342, and the intracellular fluorescence was observed by CLSM (Carl Zeiss LSM880).

### Lysosome Escape

4T1 cells (2.5 × 10^5^) were seeded in confocal dishes and cultured overnight. The cells were treated with VP‐Torin1 NPs for 1 h. Subsequently, the cells were irradiated with an Xe lamp (690 nm, 2.7 mW cm^−^^2^, 2 min) and incubated with VP‐Torin1 NPs for another 3 h. The cells at 1 or 4 h time points with or without light irradiation were cultured in an FBS‐free DMEM medium with 40 nm Lysotracker for 30 min. Afterward, cells were washed with PBS three times and incubated with Hoechst 33342 in PBS buffer for 5 min. After washing three times with PBS, the intracellular fluorescence was observed by CLSM (Carl Zeiss LSM880).

### RNA Extraction and Reverse Transcription‐PCR

HUVEC cells (8 × 10^4^) were seeded in 12‐well and cultured overnight. The cells were treated with DMSO, VP‐Torin1 NPs, or equivalent Verteporfin for 24 h. After washing with PBS, total RNA was extracted using a total RNA extraction kit (Takara 9108) according to the instructions. Then total RNA was reverse‐transcribed using PrimeScript RT reagent kit (Takara RR037A) for cDNA production by a thermal cycler (Applied Biosystems, Thermo Fisher). The amplification of YAP downstream genes (CTGF, Cyr61, EGFR, AREG) and a control gene (GAPDH) was analyzed by quantitative polymerase chain reaction (qPCR) according to the instruction (Takara RR039A) via LightCycler 480 system (Roche diagnostics). The sequences of relevant primers were as follows: CTGF, forward 5′‐CTTGCGAAGCTGACCTGGAAGA‐3′, reverse 5′‐CCGTCGGTACATACTCCACAGA‐3′. Cyr61, forward 5′‐GGAAAAGGCAGCTCACTGAAGC‐3′, reverse 5′‐GGAGATACCAGTTCCACAGGTC‐3′. EGFR, forward 5′‐AACACCCTGGTCTGGAAGTACG‐3′, reverse 5′‐TCGTTGGACAGCCTTCAAGACC‐3′. GAPDH, forward 5′‐TGGGATGGACTGTGGTCATGAG‐3′, reverse 5′‐ACTGGCGTCTTCACCACCATGG‐3′.

### Western Blot Analysis

Cells were seeded in 6‐well and cultured overnight. Then cells were incubated with DMSO or indicated treatment for 4 h. Subsequently, the cells were washed with PBS three times, cultured in fresh media, and irradiated with Xe lamp (690 nm, 2.7 mW cm^−^^2^, 90 s). After 24 h, the cells were washed with PBS and collected with RIPA buffer (supplemented with Halt protease and phosphatase inhibitor cocktail, Thermo Fisher). Protein concentration was measured by BCA kit (Thermo Fisher 23225) and the intracellular protein expression level (p‐Akt, Akt, p‐S6K, S6K, LC3, β‐actin) was analyzed by polyacrylamide gel electrophoresis. For the anti‐angiogenesis study, HUVEC cells were seeded in 6‐well and cultured overnight. Then cells were incubated with DMSO or indicated treatment for 24 h, followed by PBS washing and RIPA collection. Protein concentration was measured by BCA kit and the intracellular protein expression level (p‐Akt, Akt, p‐S6K, S6K, β‐actin) was analyzed by polyacrylamide gel electrophoresis.

### Wound Healing Assay

HUVEC cells (2 × 10^5^) were seeded in 24‐well and cultured overnight. One scratch was made in a straight line along the diameter of each well on the cell monolayer by 200 µL pipette tips. After washing three time with PBS, the cells were incubated with indicated formulations for 4 h. Then cells were washed with PBS and cultured in fresh media. After 12 and 24 h, the width of each scratch was observed by microscope. The inhibition ratio was calculated as follows:

(3) $$\mathrm{Wound}\;\mathrm{healing}\;\mathrm{level}\;\left(\%\right)=\frac{\mathrm{Width}\;\mathrm{of}\;\mathrm{scratch}\;\left(0\;h\right)\;-\;\mathrm{Width}\;\mathrm{of}\;\mathrm{scratch}\;\left(12\;\mathrm{or}\;24\;h\right)}{\mathrm{Width}\;\mathrm{of}\;\mathrm{scratch}\;\left(0\;h\right)}\times 100\%$$

(4) $$\mathrm{Inhibition}\;\mathrm{ratio}\;\;\left(\%\right)=\;\left(1-\frac{\mathrm{Wound}\;\mathrm{healing}\;\mathrm{level}\;\left(\mathrm{treatment}\;\mathrm{group}\right)}{\mathrm{Wound}\;\mathrm{healing}\;\mathrm{level}\;\left(\mathrm{control}\;\mathrm{group}\;\mathrm{in}\;\mathrm{the}\;\mathrm{dark}\right)}\right)\times 100\%$$

### Transwell Migration Assay

Migration assay was conducted on Millicell cell culture insert (12 mm, polycarbonate, 8.0 µm, Millipore PI8P01250). HUVEC cells (4 × 10^4^) were seeded on the upper chamber and incubated with indicated formulations in the media without FBS for 4 h. The lower chamber was filled with fresh media with 10% FBS. After 24 h, the cells were fixed with 4% paraformaldehyde (PFA) and stained with 0.1% crystal violet solution. Cells on the upper layer were softly wiped out by a cotton swab and the migrated cells were imaged by microscope. The relative migration ratio was calculated as follows:

(5) $$\mathrm{Relative}\;\mathrm{migration}\;\mathrm{ratio}\;\left(\%\right)=\frac{\mathrm{Number}\;\mathrm{of}\;\mathrm{migrated}\;\mathrm{cells}\;\left(\mathrm{treatment}\;\mathrm{group}\right)}{\mathrm{Number}\;\mathrm{of}\;\mathrm{migrated}\;\mathrm{cells}\;\left(\mathrm{the}\;\mathrm{control}\;\mathrm{group}\;\mathrm{in}\;\mathrm{the}\;\mathrm{dark}\right)}\times 100\%$$

### Tube Formation Assay

The 96‐well plate was first coated with Matrigel (1:1 diluted by DMEM medium) for 1 h at 37 °C. Then 4 × 10^4^ HUVEC cells were seeded on the Matrigel layer and incubated with the indicated treatment for 2 h. After 4 h, the tube formation structures of HUVEC cells were imaged by microscope.

### ATP Release Detection

4T1 cells (8 × 10^4^) were seeded in 24‐well plates and cultured overnight. Then the cells were treated with DMSO, Torin 1, Verteporfin, Torin 1 plus Verteporfin or VP‐Torin1 NPs for 4 h. Subsequently, the cells were washed with PBS, cultured in fresh media, and irradiated with Xe lamp (690 nm, 2.7 mW cm^−^^2^, 90 s). After 24 h, the supernatants were collected and ATP level was detected following the instruction provided in the kit.

### Immunofluorescent Staining

4T1 cells (2 × 10^5^) were seeded in confocal dishes (SPL100350) and cultured overnight. The cells were treated with DMSO, Torin 1, Verteporfin, Torin 1 plus Verteporfin or VP‐Torin1 NPs for 4 h. Then the cells were washed with PBS and cultured in fresh media with or without light irradiation (Xe lamp, 690 nm, 2.7 mW cm^−^^2^, 90 s). After 24 h, the cells were washed with PBS three times and fixed in a 4% PFA solution. Next, the cells were washed with PBS, permeabilized with 0.3% Triton X‐100, and blocked in 3% BSA in PBS for 1 h at room temperature. After incubating with primary antibodies (Anti‐CRT antibody or Anti‐HMGB1 antibody), secondary antibody (FITC‐conjugated Goat Anti‐Rabbit IgG H&L), and DAPI (Thermo Fisher D1306) according to the instruction, the cells were mounted with SlowFade Diamond Antifade Mountant (Thermo Fisher S36963). The blue and green fluorescence was observed by CLSM (Carl Zeiss LSM880).

### Animal Studies

BALB/c mice (4–6 weeks) were obtained from the Center for Comparative Medicine Research (Li Ka Shing Faculty of Medicine, The University of Hong Kong). All animals received care and experiments according to the guidelines and protocol approved by the Committee on the Use of Live Animals in Teaching and Research (CULTAR) at Li Ka Shing Faculty of Medicine (CULTAR No. 22–172). Animals were maintained at the CA‐DMB (Centre for Comparative Medicine Research). The 4T1 tumor‐bearing mouse model was established by subcutaneous injection of 1 × 10^6^ 4T1 cells into the right flank of the mice. The treatment procedures were initiated when tumor volume reached ≈100 mm^3^. Tumor volume was calculated as follows:

(6) $$\mathrm{Tumor}\;\mathrm{volume}\;\;\left(mm^{3}\right)=\frac{1}{2}\times \mathrm{length}\times \mathrm{widt}h^{2}$$

### Biodistribution Study

The tumor‐bearing mice were intravenously injected with 200 µL free Verteporfin (3 mg kg^−1^) or VP‐Torin1 NPs suspension via the tail vein. The fluorescence intensity was observed at indicated time points. After 24 h, the mice were euthanized and the heart, liver, spleen, lung, kidney, and tumor were excised for ex vivo fluorescence imaging. All the fluorescence detection was conducted by IVIS Lumina X5 (Alexa Fluor 700 channel).

### Antitumor Efficacy Study

The 4T1 tumor‐bearing mice were randomly divided into 6 groups (*n* = 5 per group): 1) PBS, 2) Free Torin 1 (0.35 mg kg^−1^), 3) Free Verteporfin (light irradiation, 3 mg kg^−1^), 4) Free Verteporfin plus Torin 1 (light irradiation), 5) VP‐Torin1 NPs, 6) VP‐Torin1 NPs (light irradiation). Different formulations were intravenously injected into the mice twice with an interval of one week during the treatment schedule. Light irradiation was conducted 4 h after injection by a laser (690 nm, 100 mW cm^−2^, 3 min). The body weight and tumor volume were measured every two days. After 14‐day treatment, the mice were euthanized to isolate tumor tissues, which were weighed and photographed. The tumor inhibition rate was calculated based on the tumor weight value in different groups. Besides tumors, the TDLNs and main organs were also harvested for further analysis.

### Immunofluorescent Assay of Tumor Tissues

Tumor tissues isolated from mice with different treatments after the endpoint were made into paraffin sections. The sections were dewaxed in xylene and gradient concentrations of ethanol step by step. Then the samples were washed with PBS, permeabilized with 0.3% Triton X‐100, and blocked in 10% BSA in PBS for 1 h at room temperature. After incubating with primary antibodies (Anti‐p62 antibody, Anti‐CD31 antibody, Anti‐CRT antibody, or TUNEL assay kit), secondary antibody (FITC‐conjugated Goat Anti‐Rabbit IgG H&L), and DAPI (Thermo Fisher D1306) according to the instruction, the samples were mounted with SlowFade Diamond Antifade Mountant (Thermo Fisher S36963) and coverslips. The blue and green fluorescence was observed by CLSM (Carl Zeiss LSM880).

### In Vivo Biosafety Evaluation

After the endpoint, the tumors and major organs were collected and made into paraffin sections. The morphology of cells from organs was observed after H&E staining by microscope. Alanine aminotransferase (ALT) and Aspartate transaminase (AST) activities in serum samples from mice were also measured by ALT and AST detection kit (Solarbio BC1550, BC1560) according to the instruction.

### Bilateral Tumor Model

To construct a bilateral 4T1 tumor‐bearing mice model, first 1 × 10^6^ 4T1 cells were subcutaneously injected into the right flank of the mice as primary tumors (day −8). After 8 days, 8 × 10^5^ 4T1 cells were subcutaneously injected into the left flank of the mice as distant tumors (day 0). The therapeutic monitoring began on day 0 when the primary tumor volume reached ≈100 mm^3^. The mice were randomly divided into 4 groups (*n* = 4 per group): 1) PBS, 2) Free Verteporfin plus Torin 1 (light irradiation, Verteporfin 3 mg kg^−1^, Torin 1 0.35 mg kg^−1^), 3) VP‐Torin1 NPs, 4) VP‐Torin1 NPs (light irradiation). Different formulations were intravenously injected into the mice on day 1 and day 9 during the treatment schedule. Light irradiation was conducted on primary tumor sites 4 h after injection by a laser (690 nm, 100 mW cm^−2^, 3 min). The body weight and tumor volume (primary and distant tumors) were measured every two days. After 16‐day treatment, the mice were euthanized to isolate primary and distant tumor tissues, which were weighed and photographed. Besides tumors, main organs were also harvested for further analysis.

### In Vivo Dendritic cell (DC) and T cell Analysis

For DC analysis, at the end of the 4T1 tumor‐bearing mice study, TDLNs were collected from the mice and digested with 1 mg mL^−1^ type IV collagenase and 0.2 mg mL^−1^ DNase I in RPMI complete medium for 30 min at 37 °C. Afterward, TDLNs were filtered through a 70 µm nylon cell strainer to acquire a single cell suspension. Then cells were washed with PBS three times and centrifuged at 300 g for 3 min. Zombie Yellow Fixable Viability Kit (Biolegend 423103) was used for Live/Dead staining. Then cells were incubated with anti‐CD45, anti‐I‐A/I‐E, anti‐CD11c, anti‐CD80, anti‐CD86 antibodies for 30 min. Then cells were washed with cell staining buffer and analyzed by flow cytometry (Agilent NovoCyte Quanteon). For tumor‐infiltrating T cell analysis, at the end of the bilateral 4T1 tumor‐bearing mice study, primary and distant tumors were collected, digested, and filtered into single cell suspension. Then cells were washed with PBS three times, centrifuged at 300 g for 3 min, and incubated with Zombie Yellow, anti‐CD45, anti‐CD3, anti‐CD4, anti‐CD8 antibodies for 30 min. Finally, the cells from primary and distant tumor tissues were washed with cell staining buffer and analyzed by flow cytometry (Agilent NovoCyte Quanteon).

### Statistical Analysis

All experiments were conducted three times or more independently (*n* ≥ 3). GraphPad Prism 8.3 software (GraphPad Software, Inc) was used for statistical data analysis. To compare the differences between the two groups, Independent‐Samples *t‐*test was adopted. To compare the differences between multiple‐group means, a one‐way analysis of variance was used. P value less than 0.05 was considered statistically significant. Results were presented as means ± SD.

## Conflict of Interest

The authors declare no conflict of interest.

## Supporting information

Supporting Information

## Data Availability

The data that support the findings of this study are available from the corresponding author upon reasonable request.
