# Supplementary material for: Integrating mTOR Inhibition and Photodynamic Therapy Based on Carrier‐Free Nanodrugs for Breast Cancer Immunotherapy
Source: Adv Healthc Mater. 2024 Sep 5;13(31):2402357. doi: 10.1002/adhm.202402357 (PMC11650419; doi:10.1002/adhm.202402357)
Supplement: Supplementary file 1 — Supporting Information [file ADHM-13-0-s001.docx]

Supporting Information

**Integrating mTOR inhibition and photodynamic therapy based on carrier-free nanodrugs for breast cancer immunotherapy**

*Jinzhao Liu, Qingyang Lyu, Meicen Wu, Yang Zhou, Tianyi Wang, Yichi Zhang, Ni Fan, Chang Yang, Weiping Wang**

Jinzhao Liu, Qingyang Lyu, Meicen Wu, Yang Zhou, Tianyi Wang, Yichi Zhang, Ni Fan, Chang Yang, Weiping Wang

State Key Laboratory of Pharmaceutical Biotechnology, The University of Hong Kong, Hong Kong, China

Department of Pharmacology and Pharmacy, Li Ka Shing Faculty of Medicine, The University of Hong Kong, Hong Kong, China

Dr. Li Dak-Sum Research Centre, The University of Hong Kong, Hong Kong, China

E-mail: wangwp@hku.hk

| **Name** | **Cat #** | **Fluorophore** | **Company** |
| --- | --- | --- | --- |
| Anti-p-Akt antibody | 4060 | N/A | Cell signaling Technology (CST) |
| Anti-Akt antibody | 4691 | N/A | CST |
| Anti-p-S6K antibody | 9234 | N/A | CST |
| Anti-S6K antibody | 2708 | N/A | CST |
| Anti-β-actin antibody | 4970 | N/A | CST |
| Anti-LC3B antibody | NB100-2220 | N/A | Novus |
| Anti-Calreticulin antibody | A1066 | N/A | ABclonal |
| Anti-HMGB1 antibody | A19529 | N/A | ABclonal |
| Anti-p62 antibody | ab91526 | N/A | Abcam |
| Anti-CD31 antibody | ab281583 | N/A | Abcam |
| Anti-rabbit IgG H&L (HRP) | ab6721 | N/A | Abcam |
| Goat Anti-Rabbit IgG H&L | ab6717 | FITC | Abcam |
| Anti-I-A/I-E antibody | 107623 | PerCP | Biolegend |
| Anti-CD11c antibody | 117321 | Pacific Blue | Biolegend |
| Anti-CD80 antibody | 104714 | APC | Biolegend |
| Anti-CD86 antibody | 105014 | PE-Cy7 | Biolegend |
| Anti-CD45 antibody | 157214 | FITC | Biolegend |
| Anti-CD3 antibody | 100221 | APC-Cy7 | Biolegend |
| Anti-CD4 antibody | 100427 | Pacific Blue | Biolegend |
| Anti-CD8 antibody | 100733 | PerCP-Cy5.5 | Biolegend |

**Table S1:** Antibodies used in this work for Western blot, immunofluorescence, and flow cytometry experiments.


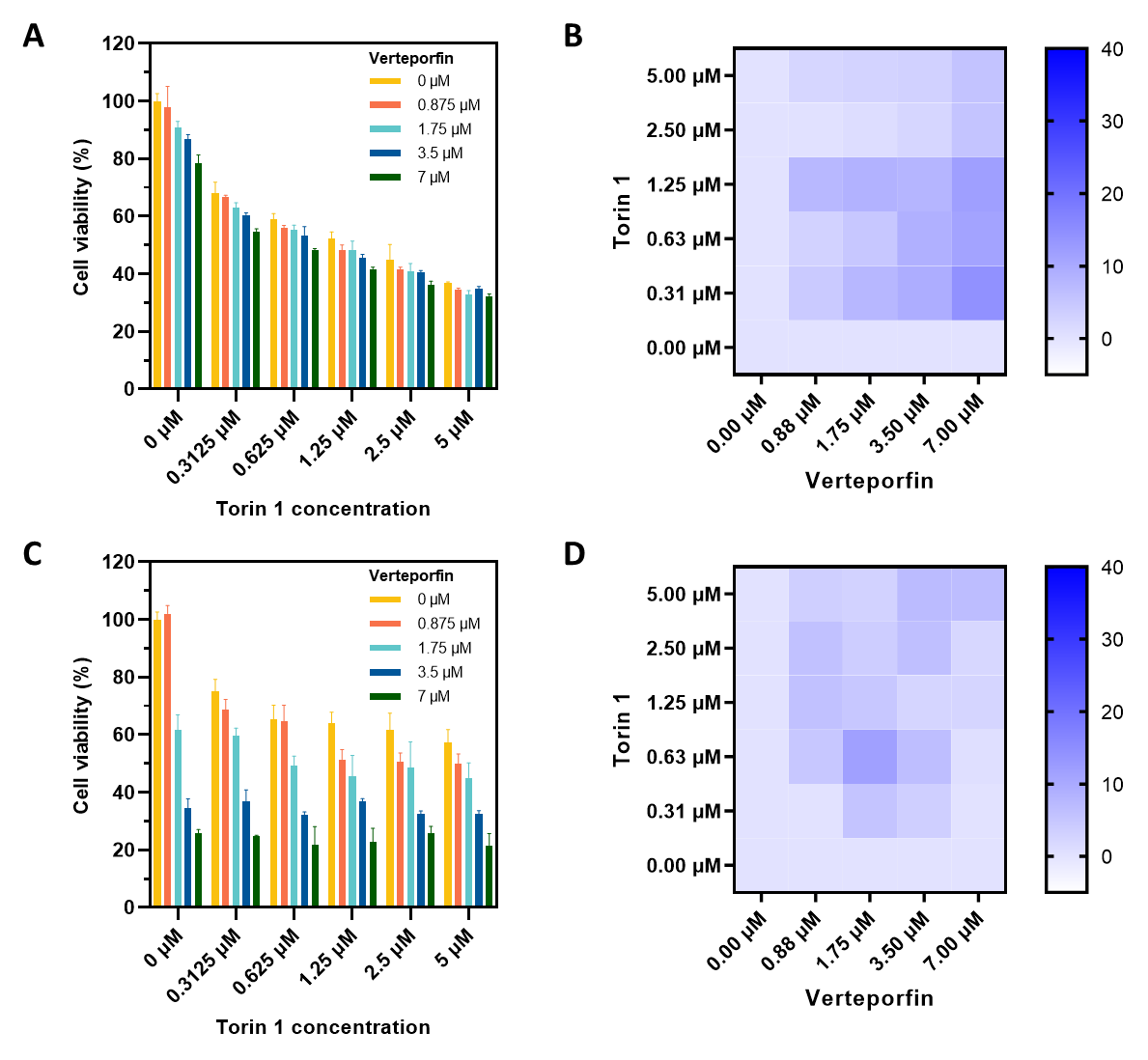


**Figure S1:** (A,C) Cell viability of 4T1 cells (A) and MDA-MB-231 (C) cells after treatment of gradient concentrations of Verteporfin and Torin 1 in the absence of light irradiation. (B,D) Synergy score of Verteporfin and Torin 1 combination on 4T1 cells (B) and MDA-MB-231 cells (D) in the absence of light irradiation calculated by HSA model in SynergyFinder.


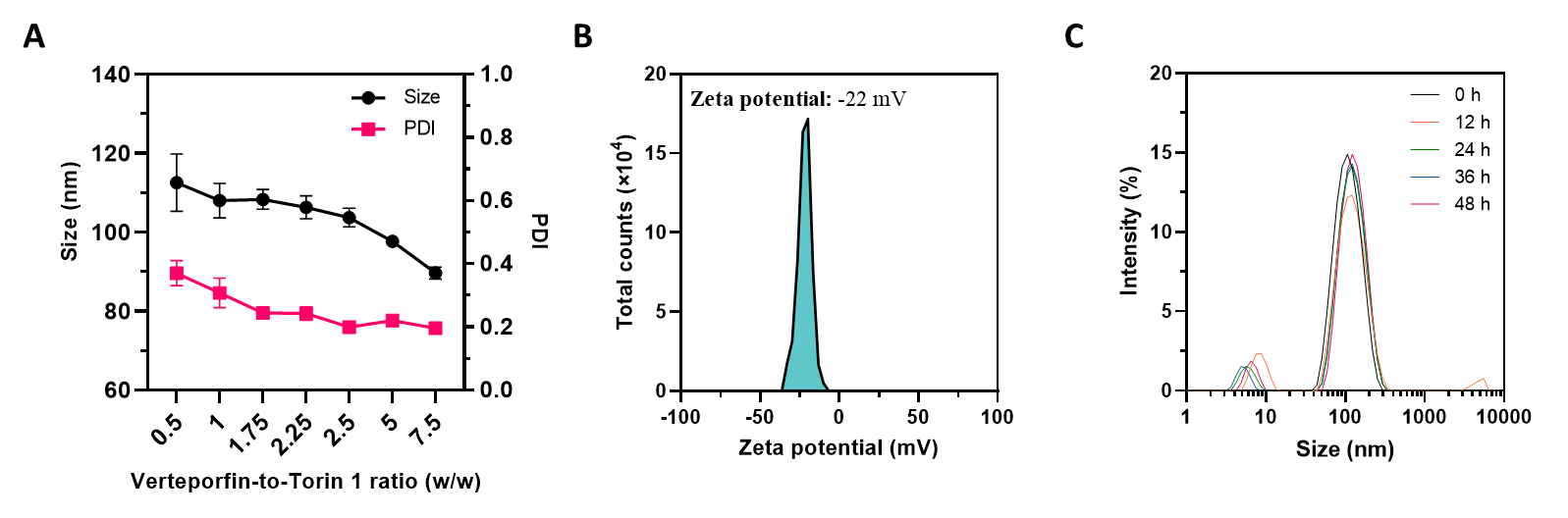


**Figure S2:** (A) Hydrodynamic sizes and PDI values of VP-Torin1 NPs at various feeding ratios. (B) Zeta potential distribution of VP-Torin1 NPs at the weight ratio of 2.5:1 characterized by DLS. (C) Stability test of VP-Torin1 NPs in FBS-contained DMEM medium at 37 ^o^C for 2 days.


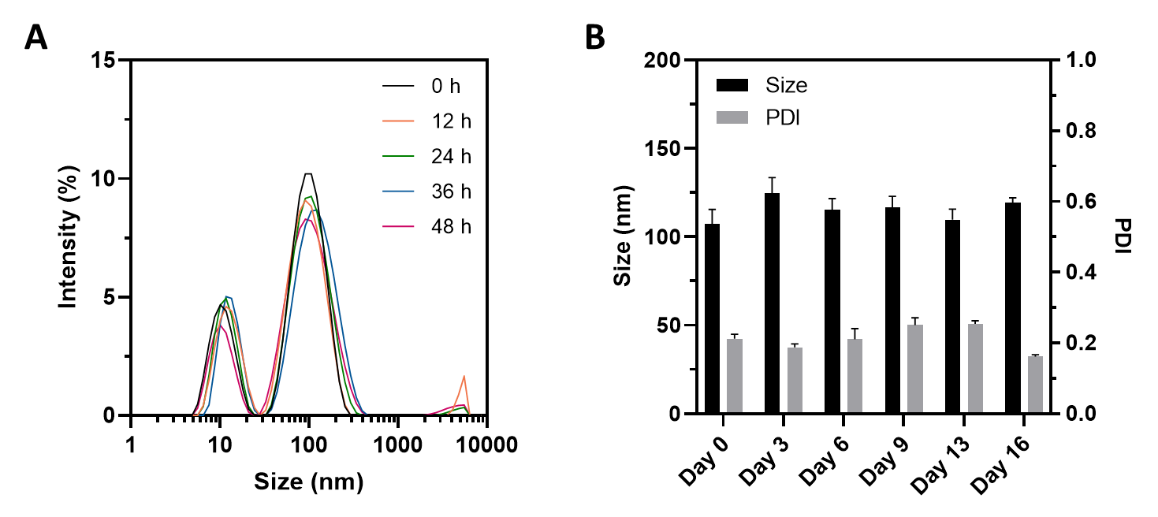


**Figure S3:** (A) Stability test of VP-Torin1 NPs in FBS at 37 ^o^C for 2 days (FBS peak was at around 10 nm and VP-Torin1 NPs’ peak was at around 100 nm). (B) Long-term stability test of VP-Torin1 NPs in the aqueous solution at 4 ^o^C for 16 days.


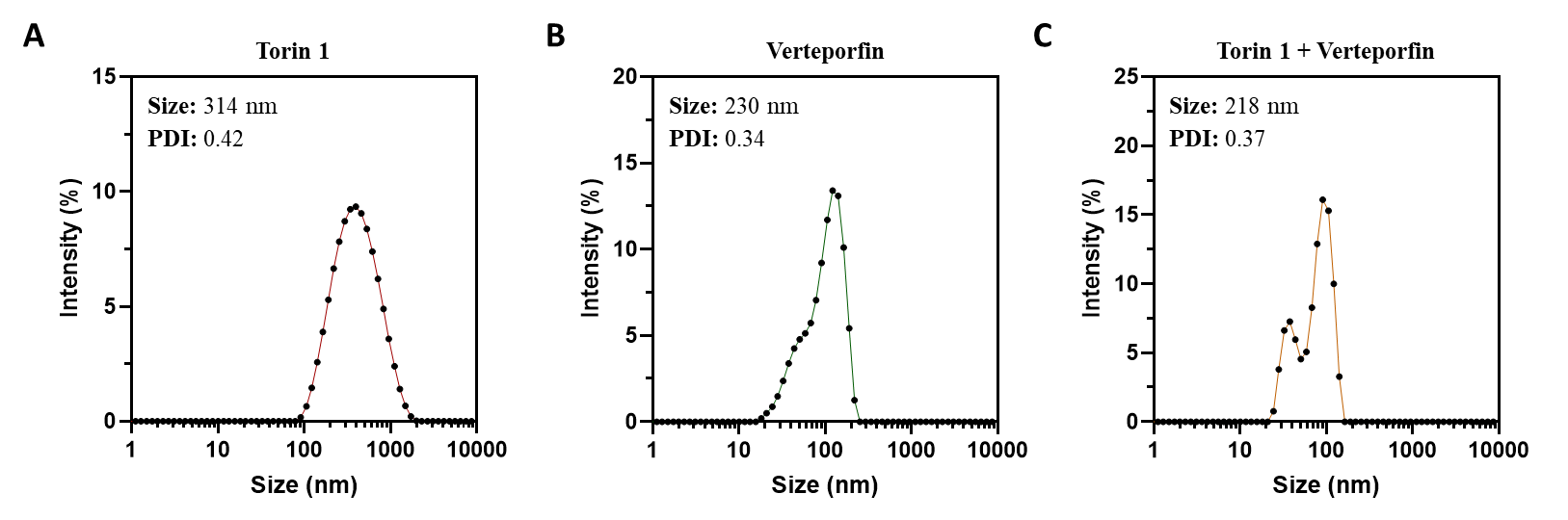


**Figure S4:** Size distribution of solutions from self-assembly of Torin 1 (A), self-assembly of Verteporfin (B), simple mixture of Torin 1 and Verteporfin (C) characterized by DLS.


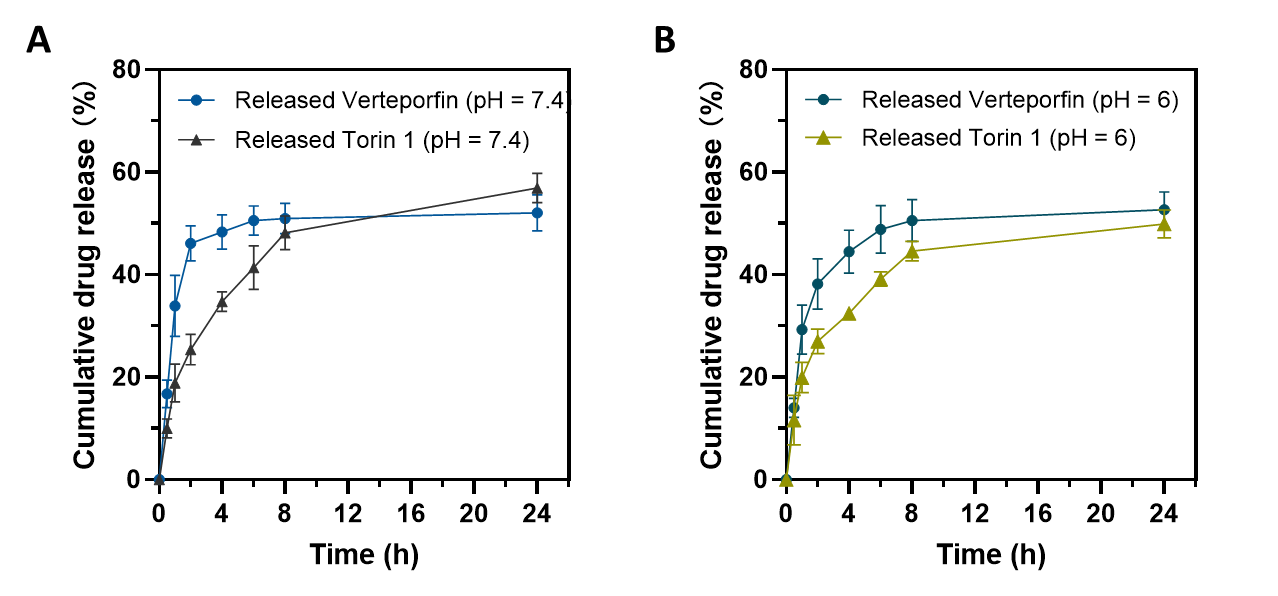


**Figure S5:** Drug release profiles of Verteporfin and Torin 1 from VP-Torin1 NPs within 24 h under the pH value of 7.4 (A) and 6.0 (B).


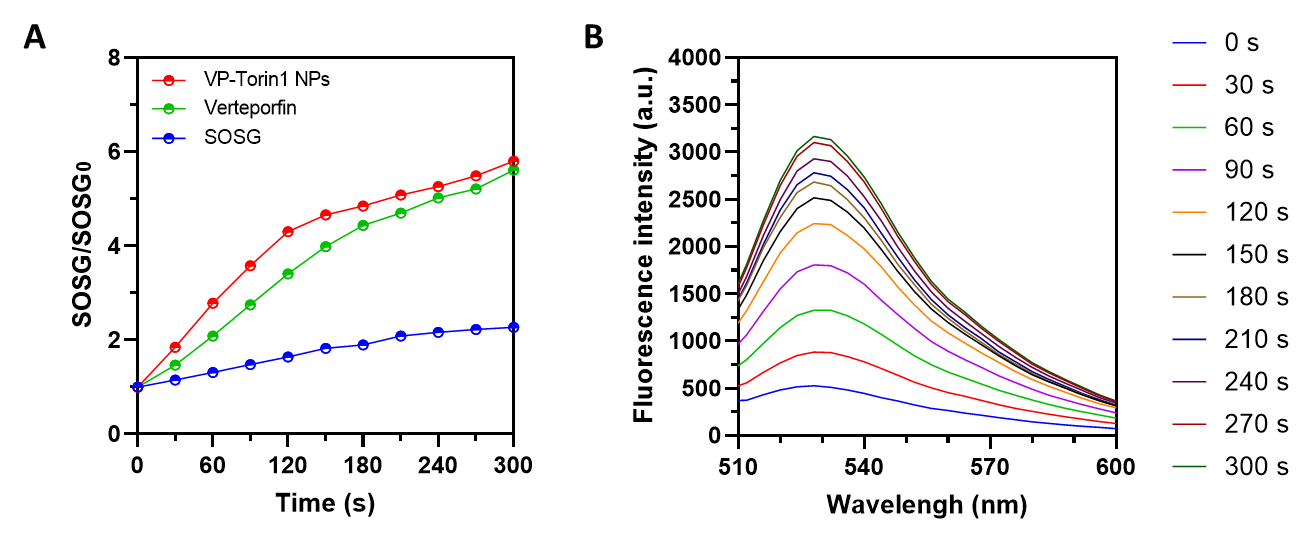


**Figure S6:** (A) The ^1^O_2_ production of VP-Torin1 NPs and free Verteporfin in the presence of light. (B) Fluorescence changes of VP-Torin1 NPs solution using SOSG as ^1^O_2_ sensor (Laser, 690 nm, 50 mW/cm², Excitation wavelength: 488 nm)


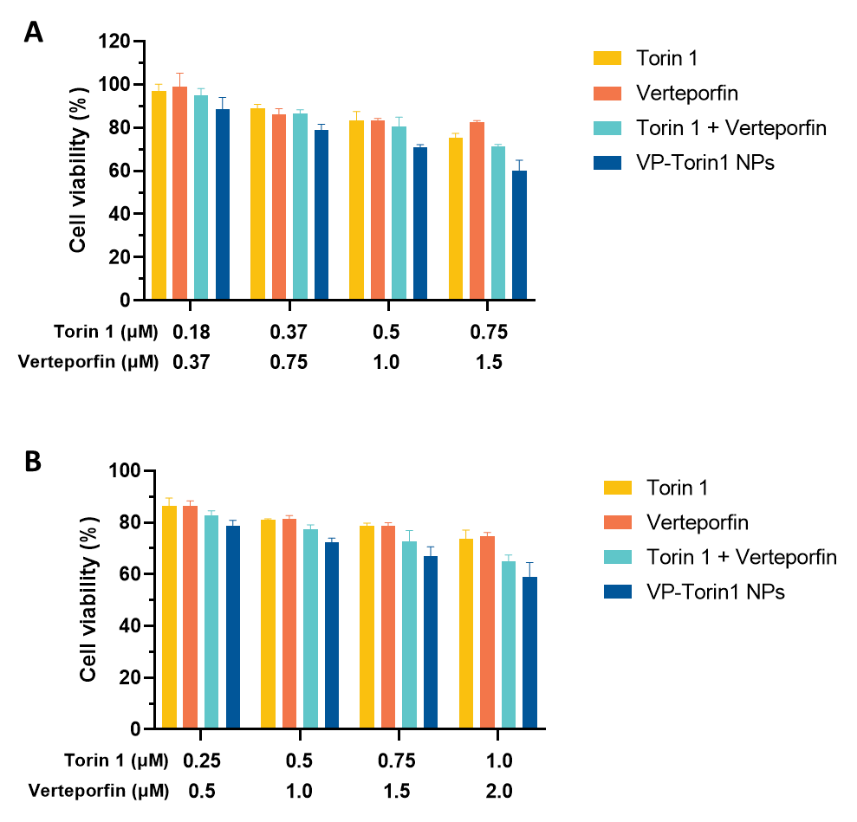


**Figure S7:** Cell viability of 4T1 cells (A) and MDA-MB-231 cells (B) after the treatment of gradient concentration of Torin 1, Verteporfin, Torin 1 plus Verteporfin, or VP-Torin1 NPs in the absence of light irradiation.

**Figure S8:** Quantitative apoptotic cell analysis of 4T1 cells after the treatment of Torin 1, Verteporfin, Torin 1 plus Verteporfin, or VP-Torin1 NPs with or without light irradiation (Xe lamp, 690 nm, 2.7 mW/cm², 2 min)


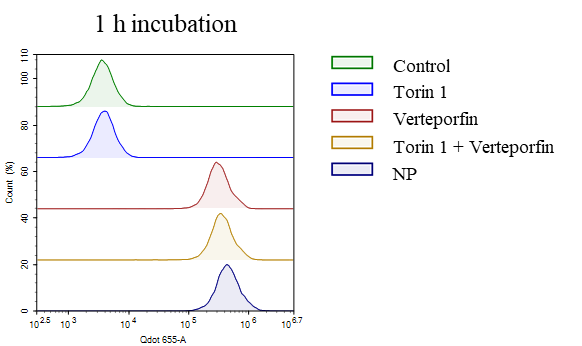


**Figure S9:** Flow cytometric analysis of cellular uptake behavior in 4T1 cells treated with Torin 1, Verteporfin, Torin 1 plus Verteporfin, or VP-Torin1 NPs for 1 h.


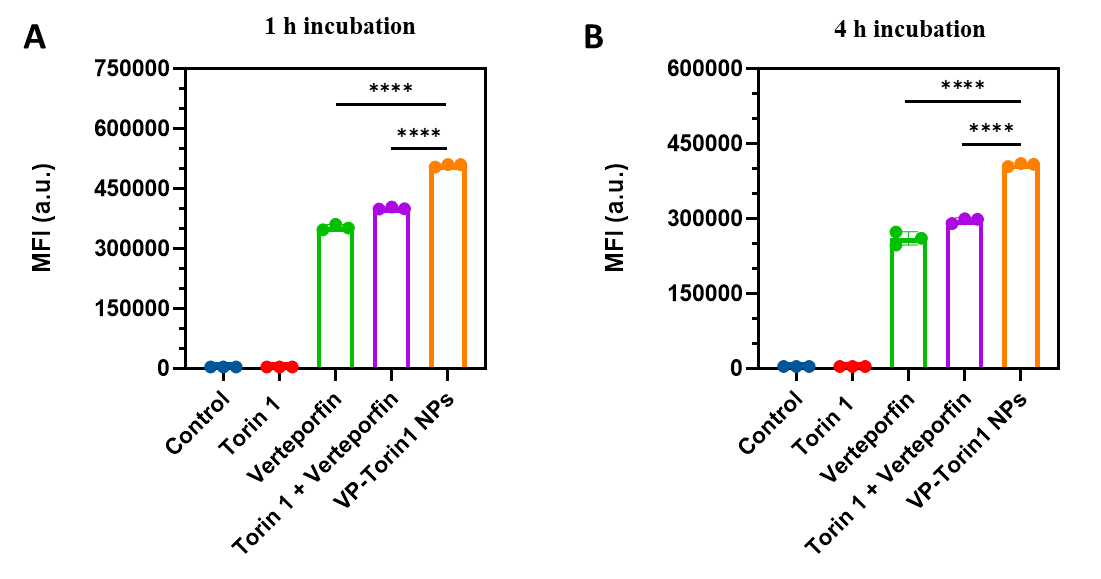


**Figure S10:** Quantitative cellular uptake analysis of 4T1 cells after treatment with Torin 1, Verteporfin, Torin 1 plus Verteporfin, or VP-Torin1 NPs for 1 h or 4 h.


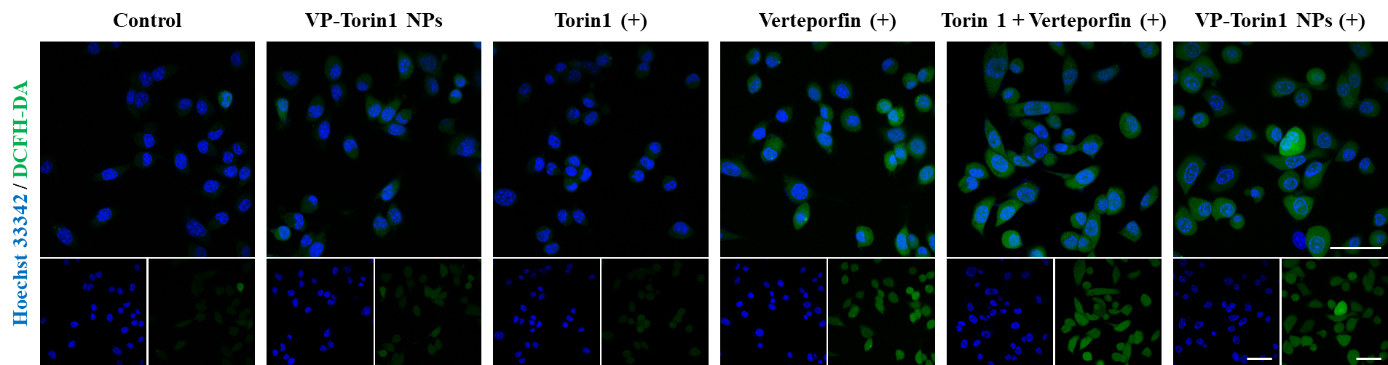


**Figure S11:** Confocal imaging assay of ROS generation inside 4T1 cells after different treatments (ROS probe DCFH-DA in green and nucleus in blue, Xe lamp, 690 nm, 2.7 mW/cm^2^, 10 min, Scale bar: 50 μm).


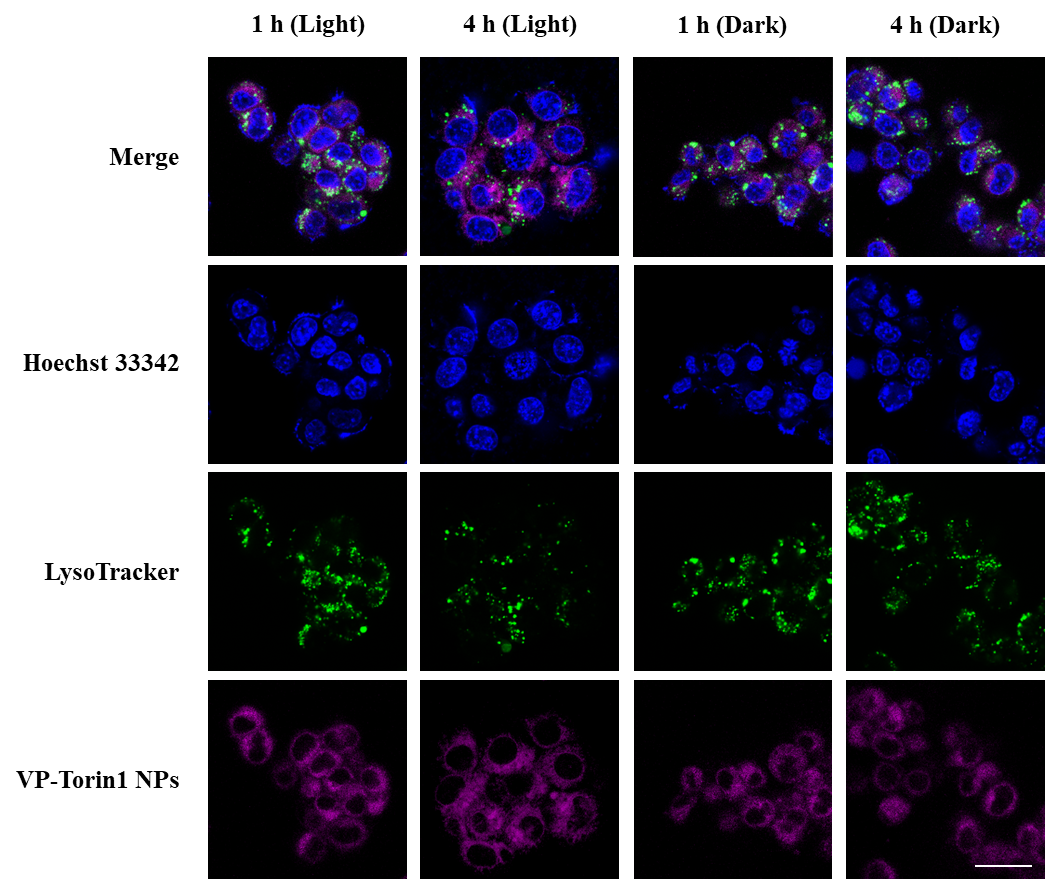


**Figure S12:** Representative confocal microscopy images of 4T1 cells incubated with VP-Torin1 NPs for 1 h or 4 h with or without light irradiation (Xe lamp, 690 nm, 2.7 mW/cm², 2 min, Scale bar: 25 μm).


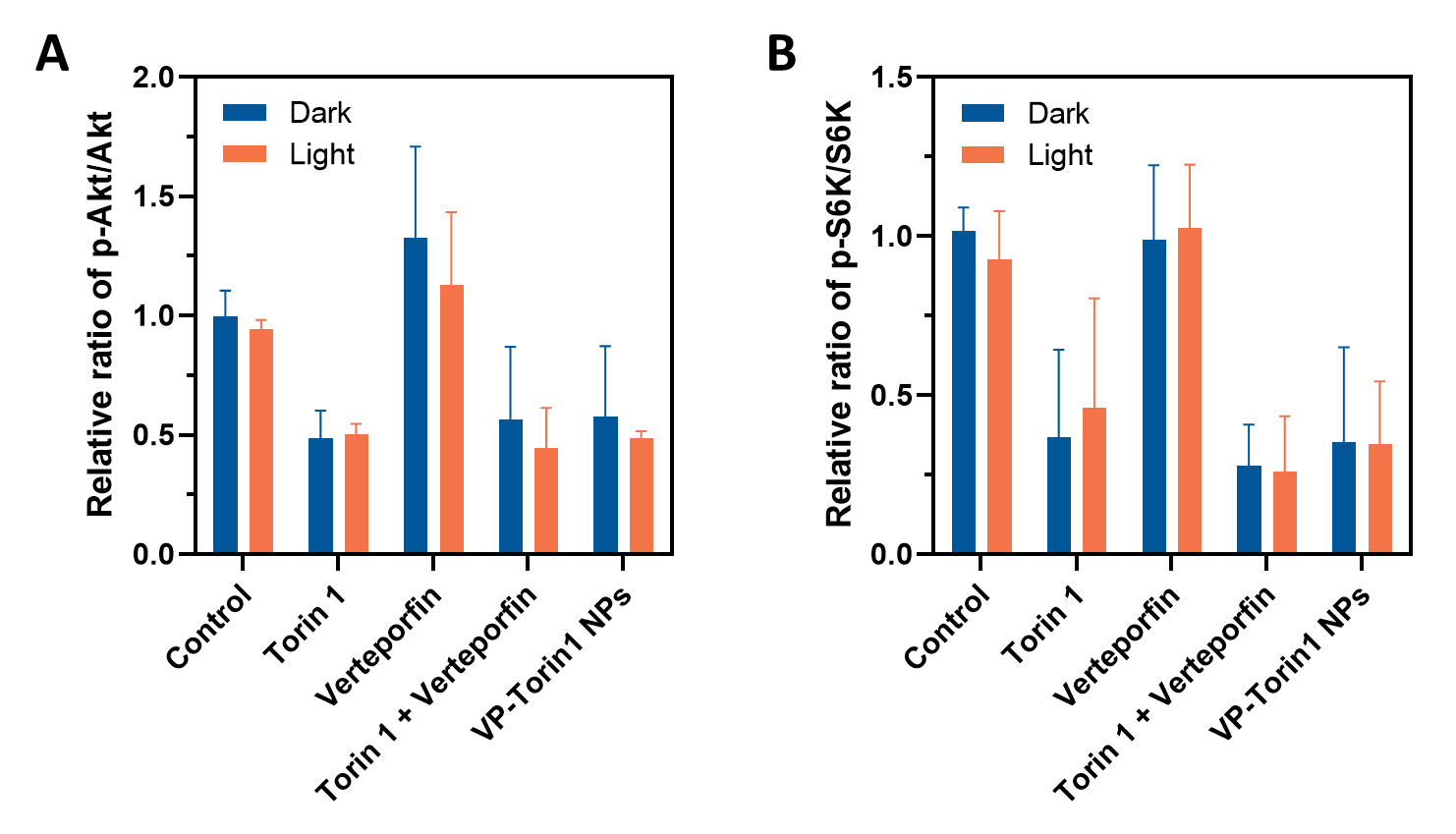


**Figure S13:** Quantitative Western Blot analysis of p-Akt / Akt (A) and p-S6K / S6K (B).


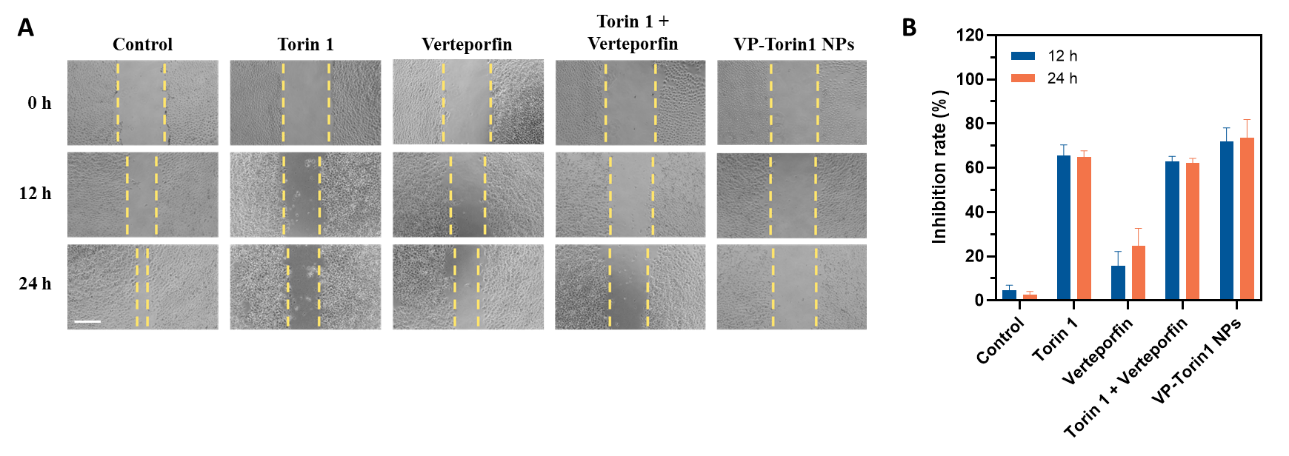


**Figure S14:** (A) Wound healing ability of HUVEC cells treated with Torin 1, Verteporfin, Torin 1 plus Verteporfin, or VP-Torin1 NPs after 12 h or 24 h (Scale bar: 0.5 mm). (B) Quantitative wound healing analysis of HUVEC cells at 12 or 24 h.

**Figure S15:** Quantitative transwell analysis of HUVEC cells.


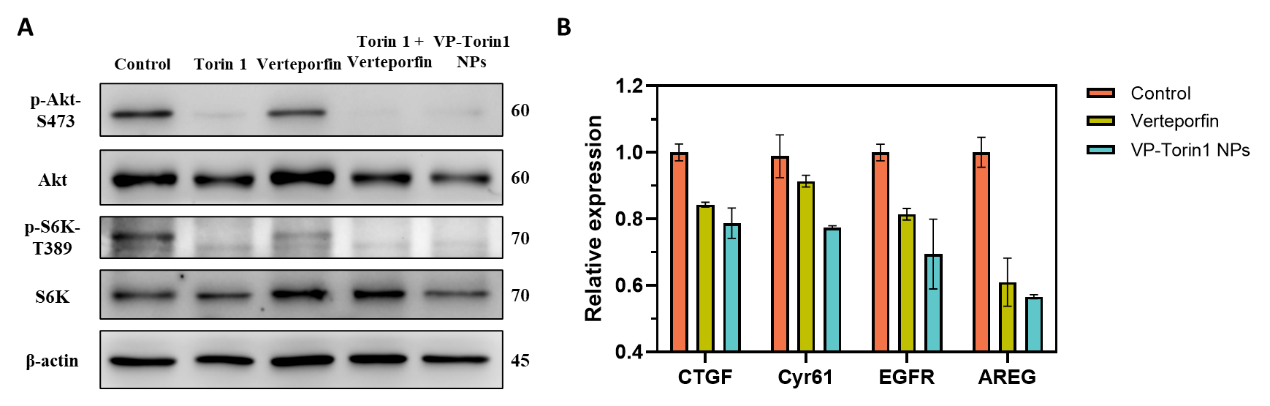


**Figure S16:** (A) Western blot analysis of mTOR downstream targets in HUVEC cells at 24 h after treatment with Torin 1, Verteporfin, Torin 1 plus Verteporfin, or VP-Torin1 NPs. (B) mRNA expression level of YAP downstream signaling pathways in HUVEC cells at 24 h after treatment with Verteporfin or VP-Torin1 NPs.


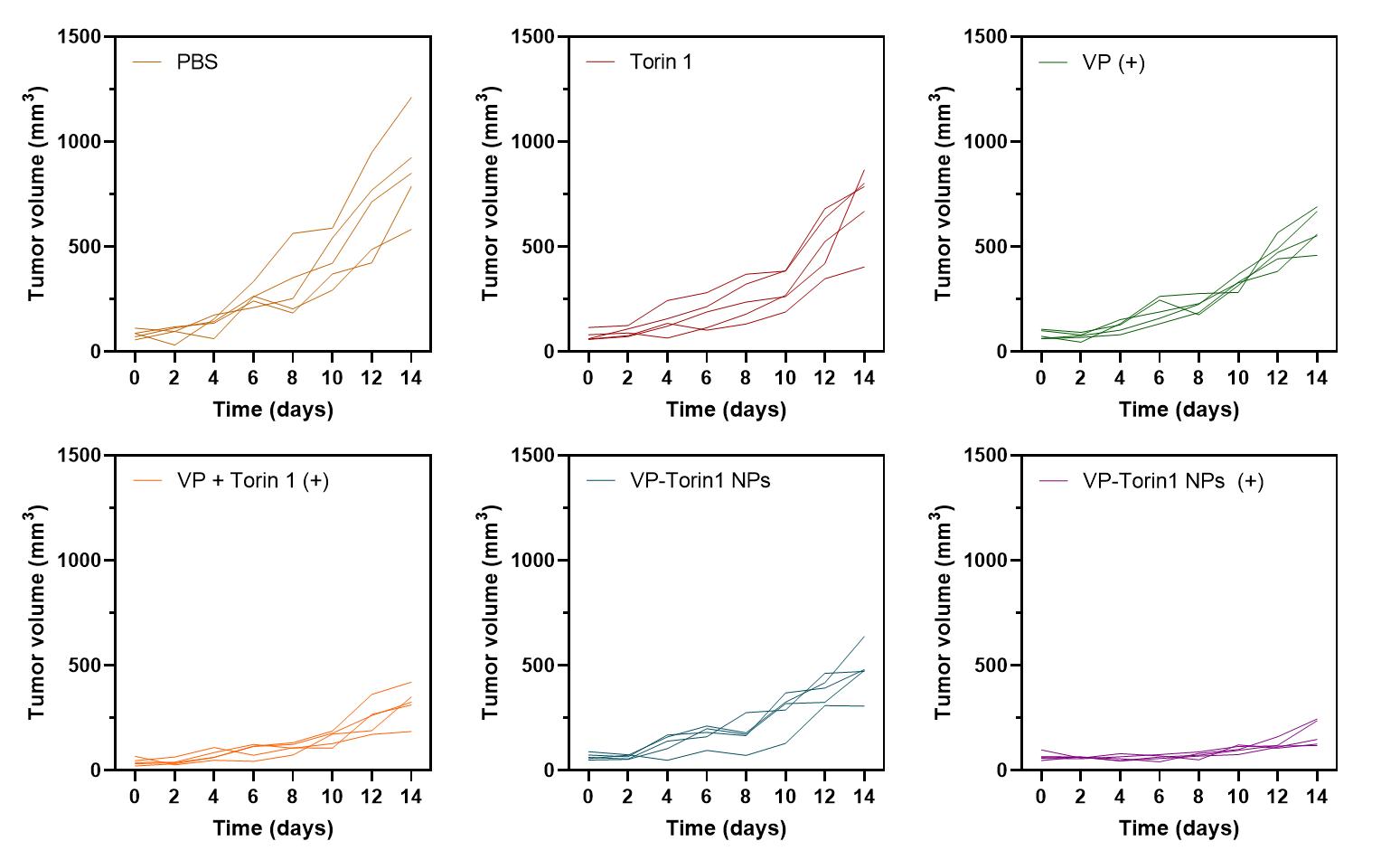


**Figure S17:** Tumor volume growth profile of individual mouse after different treatments.

**Figure S18:** Tumor inhibition rate of different groups after different treatments.


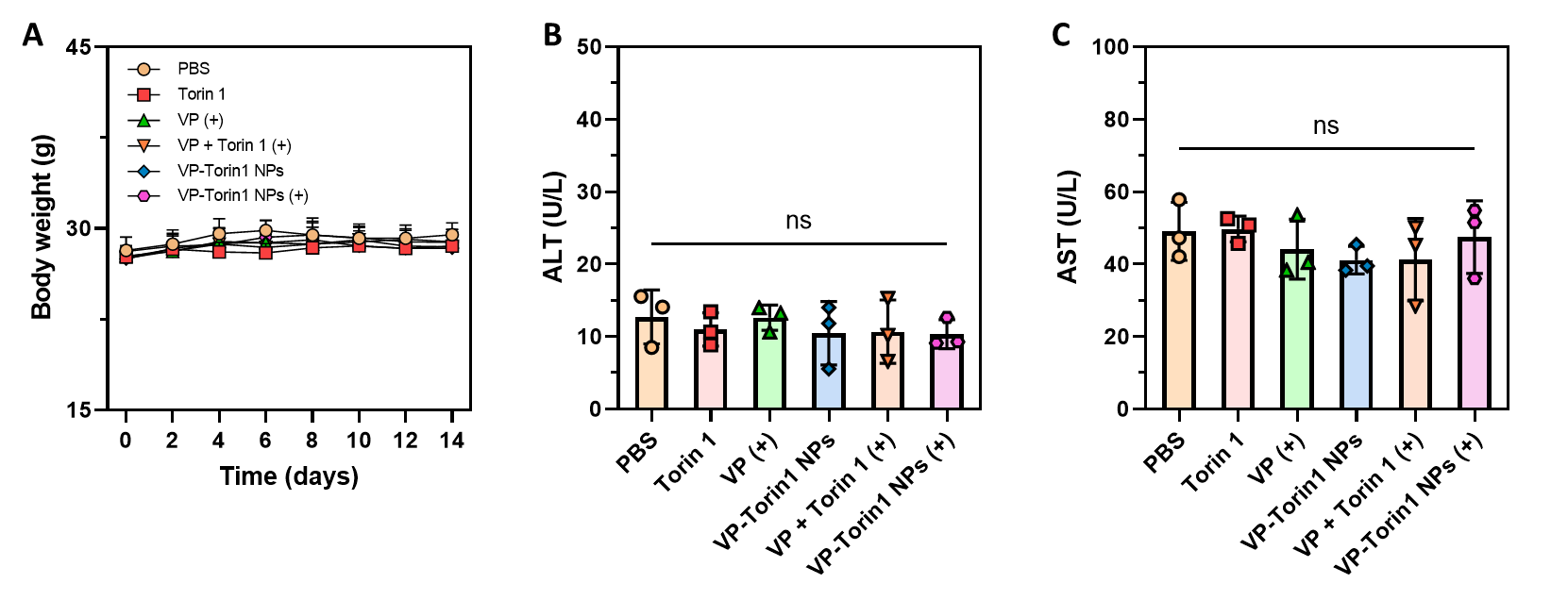


**Figure S19:** (A) Body weight profile of the 4T1 tumor-bearing mice receiving different treatments during 14-day treatment. (B,C) ALT and AST activity analysis of the serum samples from the mice treated with different formulations.


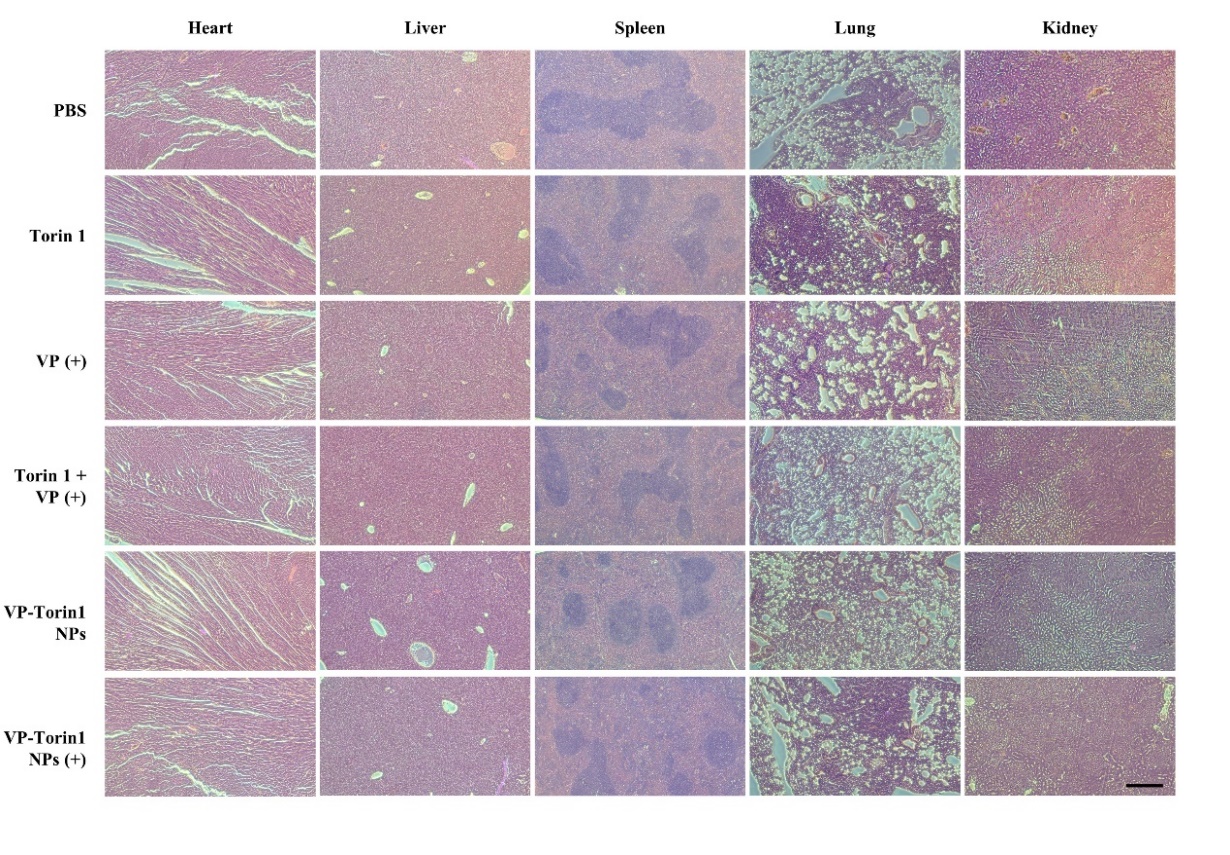


**Figure S20:** H&E staining assessment of major organs (heart, liver, spleen, lung, and kidney) from the 4T1 tumor-bearing mice after different treatments (scale bar: 0.5 mm).


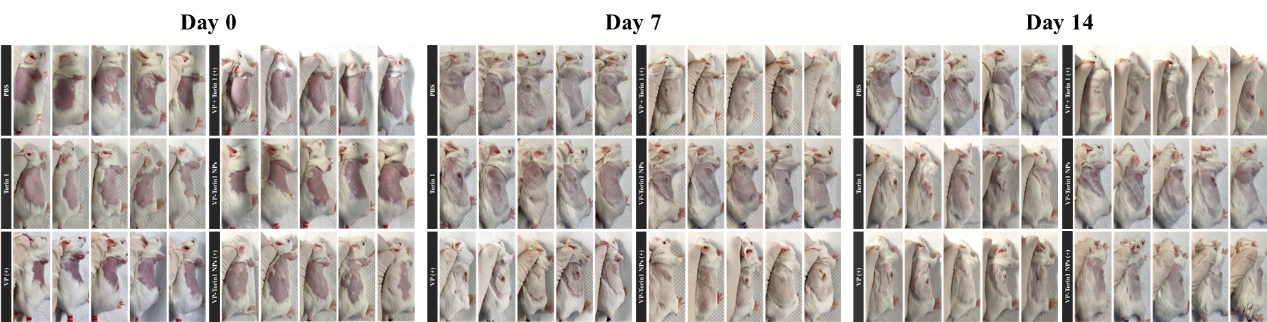


**Figure S21:** Representative photographs of the 4T1 tumor-bearing mice on day 0, day 7, and day 14 during 14-day treatment.

**Figure S22:** Body weight profile of the bilateral 4T1 tumor-bearing mice receiving different treatments during 16-day treatment.
